# Broad host tropism of ACE2-using MERS-related coronaviruses and determinants restricting viral recognition

**DOI:** 10.1038/s41421-023-00566-8

**Published:** 2023-06-15

**Authors:** Chengbao Ma, Chen Liu, Qing Xiong, Mengxue Gu, Lulu Shi, Chunli Wang, Junyu Si, Fei Tong, Peng Liu, Meiling Huang, Huan Yan

**Affiliations:** State Key Laboratory of Virology, Institute for Vaccine Research and Modern Virology Research Center, College of Life Sciences, TaiKang Center for Life and Medical Sciences, Wuhan University, Wuhan, Hubei China

**Keywords:** Mechanisms of disease, Molecular biology, Cryoelectron microscopy

## Abstract

Recently, two Middle East respiratory syndrome coronavirus (MERS-CoV) closely related to bat merbecoviruses, NeoCoV and PDF-2180, were discovered to use angiotensin-converting enzyme 2 (ACE2) for entry. The two viruses cannot use human ACE2 efficiently, and their host range and cross-species transmissibility across a wide range of mammalian species remain unclear. Herein, we characterized the species-specific receptor preference of these viruses by testing ACE2 orthologues from 49 bats and 53 non-bat mammals through receptor-binding domain (RBD)-binding and pseudovirus entry assays. Results based on bat ACE2 orthologues revealed that the two viruses were unable to use most, but not all, ACE2 from Yinpterochiropteran bats (Yin-bats), which is distinct from NL63 and SARS-CoV-2. Besides, both viruses exhibited broad receptor recognition spectra across non-bat mammals. Genetic and structural analyses of bat ACE2 orthologues highlighted four crucial host range determinants, all confirmed by subsequent functional assays in human and bat cells. Notably, residue 305, participating in a critical viral receptor interaction, plays a crucial role in host tropism determination, particularly in non-bat mammals. Furthermore, NeoCoV and PDF-2180 mutants with enhanced human ACE2 recognition expanded the potential host range, especially by enhancing their interaction with an evolutionarily conserved hydrophobic pocket. Our results elucidate the molecular basis for the species-specific ACE2 usage of MERS-related viruses and shed light on their zoonotic risks.

## Introduction

The coronaviruses (CoVs) associated with human emergence in the past two decades impose severe threats to human health, especially the recent COVID-19^1–4^. As important coronavirus reservoirs, bats have been identified as natural hosts of ancestors and relatives of three high-risk human betacoronaviruses (β-CoVs): SARS-CoV, SARS-CoV-2, and MERS-CoV^3,5–10^. MERS-CoV belongs to the lineage C of β-CoVs (*Merbecovirus* subgenus) with a high case-fatality rate (CFR) of 36%, according to the latest update of the MERS situation of the World Health Organization (WHO)^11^. NeoCoV and PDF-2180 are MERS-related viruses sampled in vesper bat harbor in South Africa and Southwest Uganda, respectively^12,13^. NeoCoV represents the yet-identified closest relative of MERS-CoV with ~85% whole genome nucleotide similarity^14^. However, the receptor binding domains (RBDs) in spike protein (S) of the two viruses are very different from MERS-CoV and many other merbecoviruses, suggesting that they have unique receptor usage^12,15^.

ACE2 mediates viral entry of many SARS-related CoVs (*Sarbecovirus* subgenus), such as SARS-CoV, SARS-CoV-2, and bat coronavirus RaTG13^3,16,17^. Moreover, the α-CoV NL63 and their bat relatives also engage ACE2 for entry^18^. In both cases, the viruses bind to a similar surface of the ACE2 protease domain, despite using two groups of structurally distinct RBDs^19^. Several merbecoviruses use host DPP4 as entry receptors, such as MERS-CoV, bat CoV HKU4, MjHKU4r-CoV and HKU25, whereas the receptors for many other merbecoviruses remain elusive^12,13,20–22^. Recently, we reported that NeoCoV and PDF-2180 unexpectedly engage bat ACE2 as their receptors^15^. Cryo-electron microscopy (Cryo-EM) analysis of NeoCoV or PDF-2180 RBD in complex with a bat ACE2 from *Pipistrellus pipistrellus* (Ppip) revealed a relatively small ACE2-binding surface featured by an N-glycosylation mediated protein–glycan interaction, a mode distinct from other ACE2-using viruses^15^.

Receptor recognition of coronaviruses is usually species-specific, acting as a primary barrier for interspecies transmission at the entry level^23,24^. Human emergence can occur through host jumping and adaptive antigenic drift of coronaviruses^25,26^. The order Chiroptera comprises > 1400 bat species with remarkable genetic diversity and wide geographic distribution, which is further divided into Yinpterochiroptera and Yangochiroptera suborders. Bats are hosts of hundreds of known α- and β-CoVs and are important for viral evolution^5^. ACE2 orthologues are largely conserved across mammalian species, while critical residues in viral receptor orthologues responsible for spike protein binding exhibit accelerated evolution^27,28^, resulting in species specificity in supporting coronavirus binding and entry, as reported in SARS-CoV, SARS-CoV-2, and MERS-CoV^29–31^. Notably, SARS-CoV-2 exhibits a broad host tropism with varying efficiency in using ACE2 orthologues from different bats and mammals^27,32–34^. Adaptive mutations of the RBD region can occur when viruses circulate in humans and other hosts^35–37^.

We have previously shown that NeoCoV and PDF-2180 selectively preferred ACE2 orthologues from Yangochiropteran bats, described as Yang-bats in this study, for entry, whereas spike mutations like T510F on receptor binding motif (RBM) markedly enhanced hACE2 binding affinity^15^. So far, the molecular basis of species-specific ACE2 recognition and potential host range of NeoCoV and PDF-2180 in diverse mammalian species remain unknown, hindering our understanding on the zoonotic risks of these viruses. By extensively examining the ACE2 orthologues from 102 mammalian species, we here demonstrated that NeoCoV and PDF-2180 can recognize ACE2 from a wide range of species and identified several critical host range determinants. We also showed that the cross-species transmission ability of these viruses could be further expanded through RBM mutations. Our data indicated a potentially broad host tropism of ACE2-using merbecoviruses, underscoring the necessity for ongoing viral surveillance in bats and other susceptible hosts to prevent future outbreaks.

## Results

### ACE2 orthologues from most Yin-bats are not well recognized by NeoCoV and PDF-2180

**Fig. 1:**
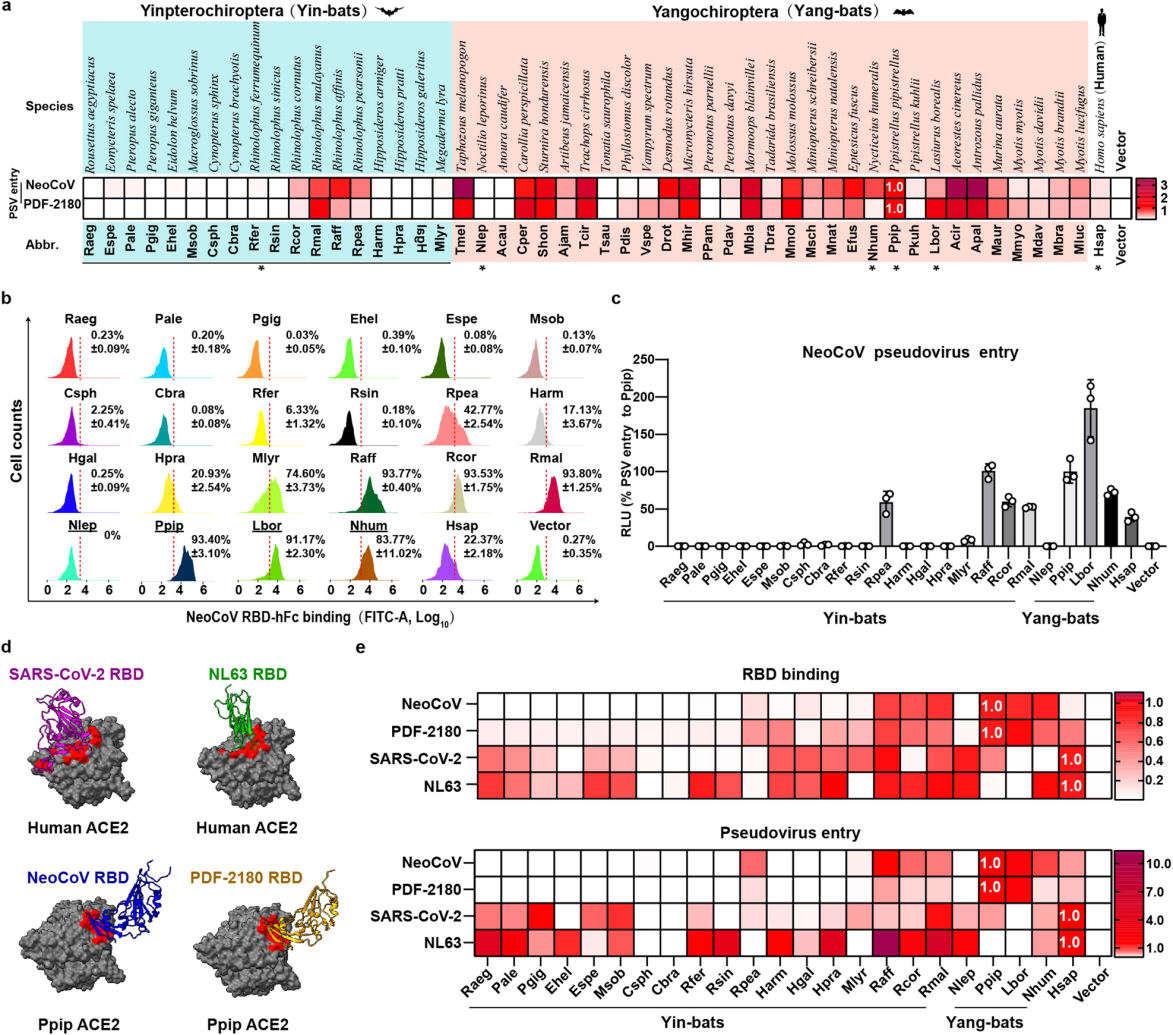
Species-specific ACE2 usage of NeoCoV, PDF-2180, and other ACE2-using viruses. **a** Heat map of pseudovirus entry efficiency (RLU relative to RLU_Ppip_) of NeoCoV and PDF-2180 in HEK293T cells stably expressing 49 bat ACE2 orthologues. * indicated ACE2 selected for subsequent characterizations. Upper, species names. Lower, abbreviations (Abbr.) of species names. Yinpterochiroptera (Yin-bats) and Yangchiroptera (Yang-bats) are indicated with cyan and pink backgrounds, respectively. The pseudovirus entry efficiency mediated by Ppip ACE2 was set as 1.0. **b**, **c** Most ACE2 orthologues from Yin-bats were deficient in supporting NeoCoV and PDF-2180 binding and pseudovirus entry. **b** Flow cytometry analysis of NeoCoV RBD-hFc binding with HEK293T cells transiently expressing the indicated ACE2 orthologues. Binding efficiency was calculated as the percentage of ACE2-expressing cells positive for RBD-Fc binding. The threshold for positive cells was determined using negative control cells transfected with empty vectors and indicated by the red dashed line. **c** Pseudovirus entry efficiency was evaluated on HEK293T cells transiently expressing the indicated ACE2 orthologues. Vector plasmid was used as a negative control. The underlines in **b** indicate species from Yang-bats. **d** Distinct RBD binding modes of four ACE2 using coronaviruses. RBD footprints of SARS-CoV-2 (PDB: 6M0J, purple), NL63 (PDB: 3KBH, green), NeoCoV (PDB: 7WPO, blue) and PDF-2180 (PDB: 7WPZ, yellow) on indicated ACE2 were highlighted in red. **e** Heat map of the RBD binding (RFU) and pseudoviruses entry efficiency (RLU) of ACE2 using coronaviruses on HEK293T cells transiently expressing the indicated ACE2 orthologues. The binding and pseudoviruses entry efficiency mediated by Ppip ACE2 was set as 1.0 for NeoCoV and PDF2180, and the values of hACE2 were set as 1.0 for SARS-CoV-2 and NL63. Representative data of three independent experiments are presented as means ± SD for *n* = 3 biologically independent cells for **b**. Data are presented as means ± SD for *n* = 3 biologically independent cells for **c**. Data are presented as means for *n* = 3 biologically independent cells for **a** and **e**. Data representative of two independent experiments. RFU relative fluorescence unit, RLU relative light units.

We previously tested the functionality of 46 bat ACE2 orthologues using a HEK293T stable cell library and found that ACE2 from most tested (14/15) Yin*-*bats, except for Rpea ACE2, was inadequate in supporting NeoCoV and PDF-2180 RBD binding and pseudovirus entry^15^. In this study, we expanded our investigation by examining the entry of NeoCoV and PDF-2180 mediated by 49 bat ACE2 orthologues stably expressed in HEK293T cells, including three new ACE2 orthologues from *Rhinolophus* bats (Rcor, Raff, and Rmal) (Fig. 1a; Supplementary Fig. S1a). Consistent with our previous report, NeoCoV and PDF-2180 showed similar receptor usage profiles and were both less capable of using ACE2 from most (14/18) Yin-bats. However, the three newly-included Yin-bats’ ACE2 were functional, suggesting that not all ACE2 from Yin-Bats were deficient in mediating viral entry of NeoCoV and PDF-2180 (Fig. 1a).

Next, NeoCoV and PDF-2180 RBD binding and entry efficiency supported by Yin-Bats’ ACE2 were verified by HEK293T cells transiently expressing ACE2 from the 18 Yin-bats, with ACE2 orthologues from four Yang*-*bats (Nlep, Ppip, Lbor, and Nhum) and hACE2 as controls (Fig. 1b, c; Supplementary Fig. S2a–c). Immunofluorescence assay detecting the C-terminal fused 3× Flag tags indicated that these receptors were expressed at a similar level (Supplementary Fig. S2d). We next investigated whether this suborder-specific bat ACE2 preference can also be observed in other ACE2-using viruses with distinct receptor binding modes (Fig. 1d). Consistent with the different RBD binding modes, SARS-CoV-2 and NL63 exhibited distinct receptor usage profiles among the 22 tested bat ACE2 orthologues as indicated by the RBD binding and pseudovirus entry assays (Fig. 1e; Supplementary Fig. S2e–h). SARS-CoV-2, which is supposed to share common ancestors infecting Yin-bats (*Rhinolophus app*.)^10^, can efficiently use 13 (> 10% to hACE2, Hsap) ACE2 orthologues from Yin-bats. Although NL63 relatives were sampled in Yang*-*bat (*Perimyotis subflavus*)^38^, most (14/18) (> 10% to hACE2, Hsap) tested Yin-bats ACE2 can support efficient binding and entry of NL63. These data indicate that the suborder-specific ACE2 usage is not strictly consistent with the phylogeny of their natural hosts, and there are specific host range determinants to be identified for ACE2-using merbecoviruses.

### NeoCoV and PDF-2180 exhibit a broad receptor tropism across non-bat mammals

**Fig. 2:**
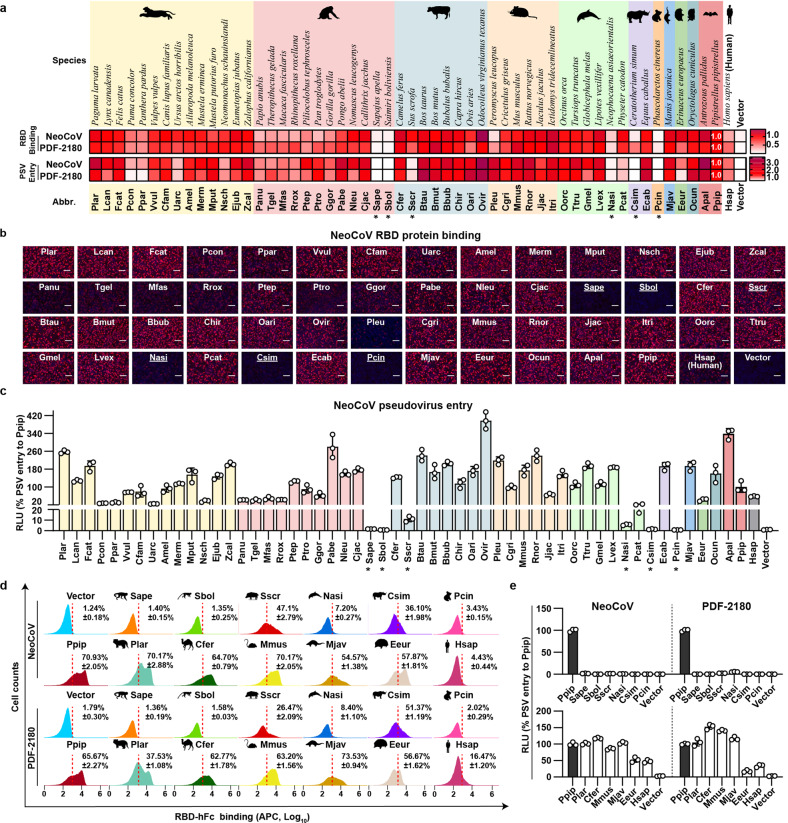
NeoCoV and PDF-2180 recognize a wide range of mammalian ACE2 orthologues. **a** The heat map of RBD binding (RFU relative to RFU_Ppip_) and pseudovirus entry efficiency (RLU relative to RLU_Ppip_) of NeoCoV and PDF-2180 mediated by various non-bat mammalian ACE2 orthologues. The pseudovirus entry efficiency and RBD binding on Ppip ACE2 were set as 1.0. *RLU < 20% RLU_Ppip_. Upper: species name. Lower: abbreviation of species name. Mammals belonging to different orders are indicated with colored backgrounds, from left to right: Carnivora, Primates, Artiodactyla, Rodentia, Cetacea, Perissodactyla, Diprotodontia, Pholidota, Erinaceomorpha, and Lagomorpha. **b** Immunofluorescence assay analyzing NeoCoV RBD-hFc binding to mammalian ACE2 orthologues transiently expressed in HEK293T cells. Scale bars, 100 μm. The six non-supportive ACE2 orthologues were underlined. **c** Entry efficiency of NeoCoV pseudoviruses in HEK293T cells transiently expressing the indicated mammalian ACE2 orthologues. *RLU < 20% RLU_Ppip_ of NeoCoV pseudovirus entry. **d** Flow cytometry analysis of NeoCoV and PDF-2180 RBD-hFc binding efficiencies with HEK293T cells transiently expressing the indicated ACE2 orthologues. The red dashed lines: the threshold for positive cells set by vector control. **e** Pseudovirus entry efficiency of NeoCoV and PDF-2180 in HEK293T cells expressing the indicated ACE2 orthologues. Data are presented as means ± SD for *n* = 3 biologically independent cells for **c**. Representative data of three independent experiments are presented as means ± SD for *n* = 3 biologically independent cells for **d**. Data are presented as means ± SEM for *n* = 3 biologically independent cells for **e**. Data representative of two independent experiments for **a**, **b**, **c**, and **e**. RLU, relative light unit.

We further explored the ability of ACE2 orthologues from 53 non-bat mammals to support NeoCoV and PDF-2180 RBD-binding and pseudovirus entry, most of which were selected and tested for SARS-CoV-2 in a previous study^27^. These species include wild and domestic animals belonging to ten mammalian orders: Carnivora, Primates, Artiodactyla, Rodentia, Cetacea, Perissodactyla, Diprotodontia, Pholidota, Erinaceomorpha, and Lagomorpha (Supplementary Fig. S1b). These mammals include animals in frequent contact with humans, model animals, and endangered animals, and some are potentially natural or intermediate hosts of coronaviruses. The expression of all ACE2 orthologues was verified by immunofluorescence (Supplementary Fig. S3a). We then conducted NeoCoV and PDF-2180 RBD immunofluorescence-based live cell binding and pseudovirus entry assays to test their receptor function by transiently expressing them in HEK293T cells, with ACE2 from Ppip bat (Ppip ACE2) as a positive control. The binding and entry assays showed generally consistent results in most species (Fig. 2a). Besides, species showing inconsistent immunofluorescence binding assay and pseudovirus entry assays were verified by flow cytometry-based binding assays (Supplementary Fig. S3b). 47 out of 53 ACE2 orthologues could support considerable entry of both viruses, albeit with varying entry efficiencies (> 20% to Ppip ACE2 for NeoCoV). Several primates, including humans, showed relatively low efficiency to support RBD binding and entry of both viruses. The six ACE2 orthologues showing undetectable or very limited entry (< 20% to Ppip ACE2 for NeoCoV) were from five different orders: Sape (*Sapajus apella*), Sbol (*Saimiri boliviensis*), Sscr (*Sus scrofa*), Nasi (*Neophocaena asiaeorientalis*), Csim (*Ceratotherium simum*), and Pcin (*Phascolarctos cinereus*) (Fig. 2a). Representative RBD binding and entry efficiency of NeoCoV and PDF-2180 were shown (Fig. 2b, c; Supplementary Fig. S3c, d). Next, we further assessed the NeoCoV RBD binding and entry efficiency with ACE2 orthologues from the above species and with six natural or intermediate host species for β-coronaviruses as controls^39–43^. The expression levels of the selected ACE2 orthologues were verified by Western blot assay (Supplementary Fig. S3e, f). As expected, the RBD binding and pseudovirus entry data confirmed that the ACE2 from the six β-CoV host-related species showed entry-supporting capability compared to the six inadequate ACE2 orthologues (Fig. 2d, e; Supplementary Fig. S3g, h). Collectively, these data demonstrate that ACE2 orthologues from a wide range of species can function as receptors for NeoCoV and PDF-2180, suggesting that these species might be susceptible to these viruses.

### Identification of host tropism determinants restricting NeoCoV and PDF-2180 recognition

**Fig. 3:**
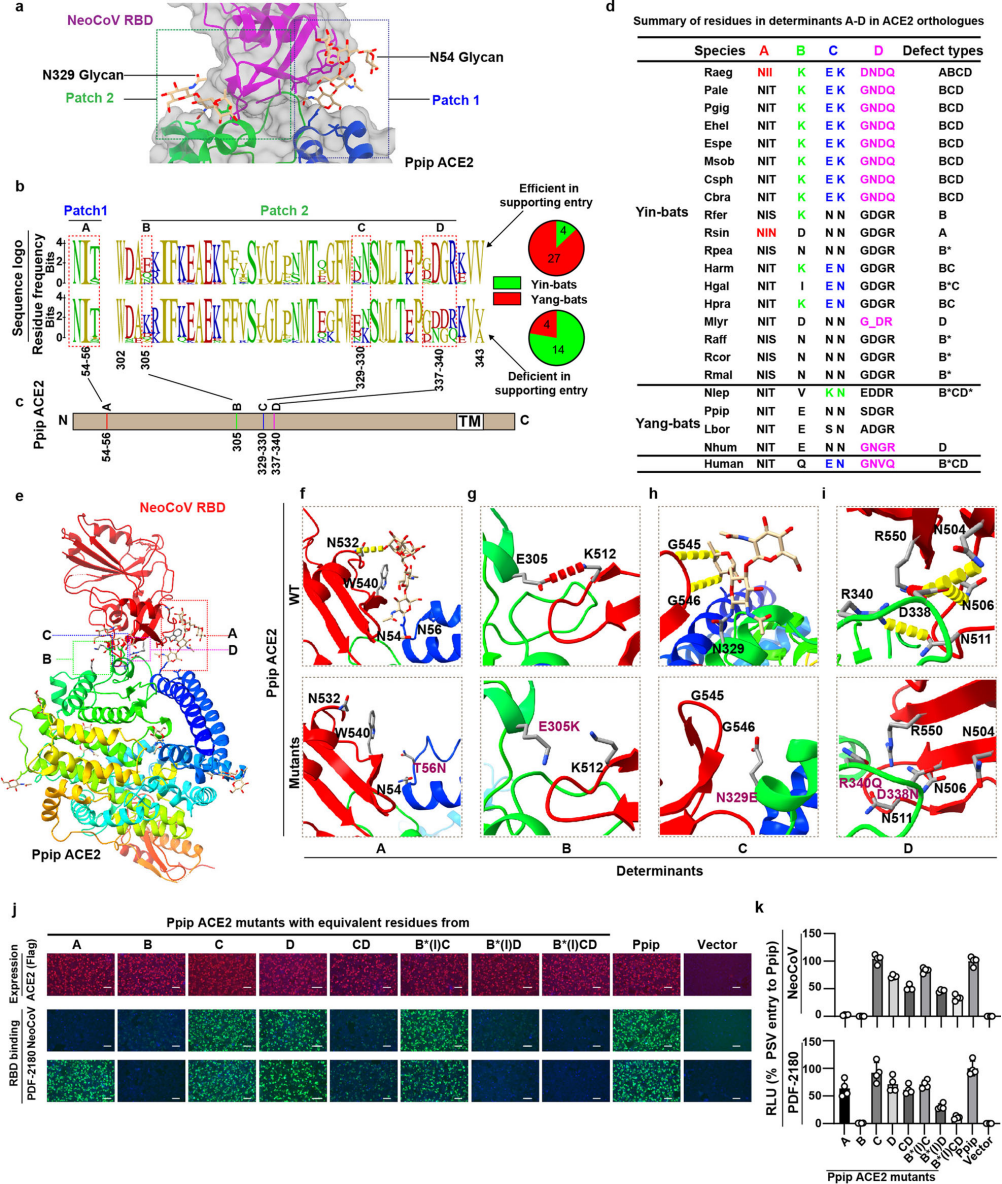
Identification of host range determinants restricting NeoCoV and PDF-2180 recognition. **a** Magnified view of the binding interface between NeoCoV RBD (purple) and Ppip ACE2 (rainbow) (PDB:7WPO). Patch 1 and patch 2 indicate two main interaction regions, each containing a glycosylation on Ppip ACE2. **b**, **c** Comparative sequence analysis predicting the potential host range determinants. **b** Residue conservation of the two critical virus-binding loops based on sequences of 49 ACE2 orthologues, which are separated into two groups according to their entry-supporting efficiency. Upper: efficient in supporting NeoCoV and PDF-2180 entry (> 10% RLU_Ppip_). Lower: deficient in supporting NeoCoV and PDF-2180 entry (< 10% RLU_Ppip_). The pie charts summarize the numbers of Yin-bats and Yang-bats in each group. **c** The four variable regions showing group-specific residue frequencies were defined as determinants A–D. Sequence numbers were based on Ppip ACE2. TM, transmembrane motif. N: N-terminus, C: C-terminus. **d** Summary of defect types of bat and human ACE2 orthologues according to their residue features in determinants A–D. The predicted sub-optional residues in determinants A (54–56), B (305), C (329–330), and D (337–340) were highlighted with red, green, blue, and magenta, respectively. *Determinants with sub-optional but acceptable sequences. **e**–**i** Structural analyses of the impact of residue substitutions corresponding to Yin bats’ ACE2 on the interaction between Ppip ACE2 and NeoCoV RBD. **e** Structure of Ppip ACE2 and NeoCoV RBD complex (PDB:7WPO), with each determinant indicated by dashed boxes. **f**–**i** Magnified view of the interface of determinants A–D according to the WT (upper) and mutated (lower) Ppip ACE2 reconstructed using Coot software. All structures are shown as ribbon representations, with key residues rendered as sticks. Salt bridges and hydrogen bonds are shown as red and yellow dashed lines. Mutated residues were highlighted in purple. **j**, **k** NeoCoV and PDF-2180 RBD-hFc binding (**j**) and pseudovirus entry (**k**) in HEK293T cells transiently transfected with the mutated Ppip ACE2 carrying the representative Yin bats’ residues in determinant A, B, C, D and CD, respectively. A (N54T), B (E305K), C (N329E, N330K), D (SDGR to GNGQ) and CD, respectively. B*(I) indicates an E305I mutation. Data representative of two independent experiments for **j**. Representative data of three independent experiments are presented as means ± SD for *n* = 4 biologically independent cells for **k**. Scale bars represent 100 μm for **j**.

We next sought to identify the host tropism determinants through comparative analyses of the two critical RBD-interacting loops on ACE2 orthologues, each containing a glycosylation site in Ppip ACE2 (Fig. 3a). We first conducted multi-sequence alignment and conservation analyses based on 49 bat ACE2 sequences (from 18 Yin- and 31 Yang-bats), which were divided into two groups based on their ability to support NeoCoV entry based on the data of Fig. 1a (Fig. 3b; Supplementary Figs. S1a and S4a). While bat ACE2 orthologues are largely conserved, highly variable residues can be found within the virus-binding loops. The comparative analysis identified four candidate determinants (from A–D) with contrasting residue frequencies across the two groups, all located in the RBD-binding interface (Fig. 3c). We defined and classified the ACE2 orthologues deficient in NeoCoV and PDF-2180 receptor function into several defect types based on the presence of putatively unfavorable/sub-optimal residues (Fig. 3d). For example, Raeg ACE2 was considered defect type ABCD as it carries putatively unfavorable residues on all four determinants. Determinants containing sub-optimal but still acceptable residues were annotated with an asterisk.

We next analyzed the impact of the unfavorable/sub-optimal ACE2 residues on viral RBD recognition based on the structure of the Ppip ACE2-NeoCoV-RBD complex (Fig. 3e–i). Determinants A and C contain glycosylations sites (N-X-T/S) required for the glycan–protein interactions and are absent in several Yin-bats’ ACE2. Determinant A glycosylation (N54 glycosylation) (Fig. 3f) plays a more crucial role than determinant C glycosylation (N329-glycosylation) (Fig. 3h) for viral receptor interaction as it mediated an indispensable protein–glycan interaction underpinning the RBD binding away from the main protein–protein binding interface, especially for NeoCoV. This explains why Lbor ACE2, without a determinant C glycosylation, remains a receptor with a strong entry-supporting ability for NeoCoV and PDF-2180. Residues in determinants B (Fig. 3g) and D (Fig. 3i) of Yin-bats’ ACE2 either abolish the polar contacts (e.g., two salt bridges formed by residues E305 and D338, respectively) or introduce steric hindrance (e.g., E305K) that reduce the binding affinity. It is worth mentioning that determinant D, especially the D338, is also a critical host range determinant restricting hACE2 from efficiently supporting the binding and entry of the two viruses^15^.

We also conducted a similar comparative analysis based on ACE2 orthologues from non-bat mammals (Supplementary Fig. S5a), which is less informative than bat species as only six non-bat mammalian species are potentially non-permissive. We conducted sequence conservation analysis of non-bat ACE2 orthologues based on three groups: the total 53 non-bat mammals, 6 deficient ACE2 orthologues, and 30 competent ACE2 orthologues based on the data of Fig. 2c (NeoCoV entry supporting ability > Ppip ACE2), respectively (Supplementary Fig. S5b). Compared with the four determinants identified among bat species, the analysis of ACE2 orthologues from the six non-bat mammals mainly pointed to putatively unfavorable/sub-optional residues in determinant B (residue 305), which is associated with a loss of a salt bridge interaction as indicated by the cryo-EM structure of NeoCoV RBD–Ppip ACE2 complex (Fig. 3g)^15^.

To demonstrate the potential importance of the four predicted determinants, we generated a set of loss-of-function mutations for Ppip ACE2 by substitution with the representative Yin bats’ residues in determinants A, B, C, D, and CD, respectively. Pseudovirus entry assay and RBD binding assays were conducted to evaluate the receptor function. The result showed that only A, B, and CD exhibited a significant loss of function in pseudovirus entry or RBD binding (Fig. 3j, k; Supplementary Fig. S5c, d). We further generated loss-of-function mutants for determinants C, D, and CD loss-of-function mutants in addition to the B* mutation (E305I) of Ppip ACE2 (Fig. 3j, k), and the results confirmed the importance of C and D in the presence of sub-optimal residue in determinant B.

### Functional verification of host tropism determinants of NeoCoV and PDF-2180 in Yin-bats

**Fig. 4:**
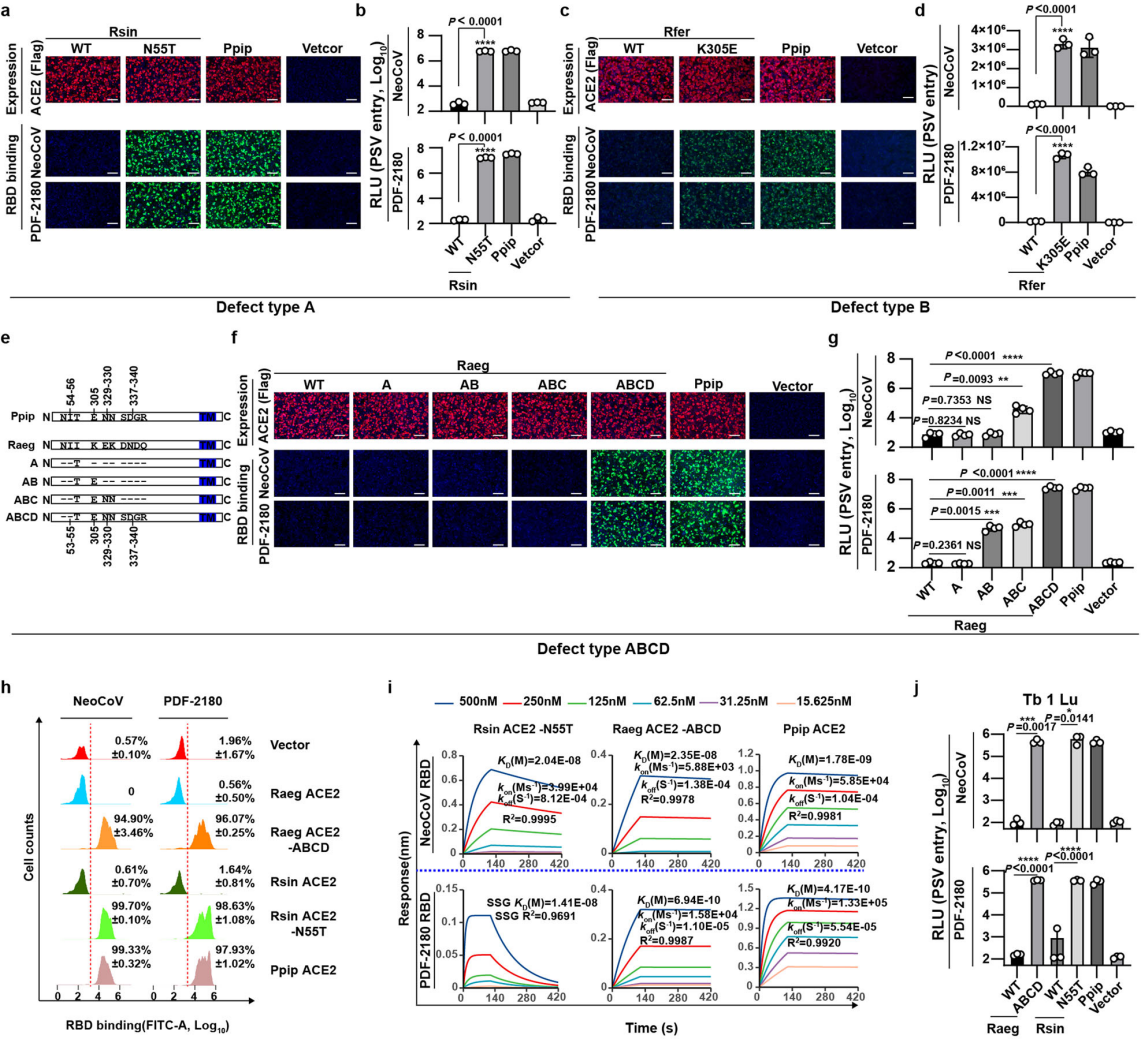
Verification of determinants crucial for species-specific receptor usage of NeoCoV and PDF-2180 in bats. **a**–**g** Gain of receptor function of Yin-bats’ ACE2 orthologues in supporting NeoCoV and PDF-2180 RBD binding (**a**, **c**, **f**) and pseudoviruses entry (**b**, **d**, **g**) through the indicated mutations. **a**, **b** Rsin ACE2 (defect type A); **c**, **d** Rfer ACE2 (defect type B); **f**, **g** Raeg ACE2 (defect type ABCD). **e** Schematic illustration of Raeg ACE2 swap mutants carrying the indicated Ppip ACE2 counterparts. Data related to defect type B*, B*C, and B*CD* can be found in Supplementary Fig. S6f–l. **h** Flow cytometry analysis of NeoCoV and PDF-2180 RBD-hFc binding efficiencies with HEK293T cells transiently expressing the indicated ACE2 orthologues. The red dashed lines: the threshold for positive cells set by vector control. **i** BLI assays analyzing the binding kinetics between NeoCoV RBD-hFc/PDF-2180 RBD-hFc and the indicated ACE2 ectodomain proteins with indicated mutations. SSG: steady-state graph affinity determination. **j** NeoCoV and PDF-2180 pseudovirus entry in Tb 1 Lu bat cells transiently expressing the indicated ACE2 orthologues at 16 h post-infection. Data are presented as means ± SD for *n* = 3 biologically independent cells for **b** and **d**, *n* = 4 for **g**. Data representative of three independent experiments for **a**–**g**. Representative data of three independent experiments are presented as means ± SD for *n* = 3 biologically independent cells for **h**. Representative data of two independent experiments are presented as means ± SD for *n* = 3 biologically independent cells for **j**. Two-tailed unpaired Student^’^s *t*-test for **b**, **d**, **g**, and **j**; *P < 0.05, **P < 0.01, ***P < 0.005, and ****P < 0.001. NS not significant. RLU Relative luciferase unit. Scale bars in **a**, **c**, and **f**: 100 μm.

To further verify the predicted determinants, we selected representative ACE2 orthologues of specific defect types for mutagenesis assays to improve their receptor function. Specifically, We generated a series of mutations based on ACE2 orthologues from Rsin (type A), Rfer (type B) and Rpea (type B*), Hgal (type B*C), Nlep (type B*CD*), and Raeg (type ABCD) for gain-of-function tests. The results showed that the N55T point mutation, which activates an N53 glycosylation site (Rsin ACE2 has a deletion on site 19 compared to Ppip ACE2), markedly improved the receptor function of Rsin ACE2 (type A) (Fig. 4a, b; Supplementary Fig. S6a). Similarly, introducing a proper side chain for salt bridge formation in Rfer-K305E (type B) and Rpea-N305E (type B*) led to significantly improved receptor function (Fig. 4c, d; Supplementary Fig. S6b–e). Efficient binding and pseudovirus entry mediated by bat ACE2 mutants from Hgal (type B*C) and Nlep (type B*CD*) were achieved after replacing corresponding residues with Ppip ACE2 counterparts (Supplementary Fig. S6f–l). Remarkably, a gradual gain of receptor function of Raeg ACE2 (type ABCD) can be observed following stepwise increased substitutions of determinants A, AB, ABC, and ABCD with Ppip ACE2 counterparts, respectively (Fig. 4e–g; Supplementary Fig. S6m). We further evaluated the binding affinities between viral RBD proteins and representative wild type (WT) and mutant ACE2 by Flow cytometry and Bio-layer interferometry (BLI) assays (Fig. 4h, i). As expected, the binding affinities between WT Raeg/Rsin ACE2 and NeoCoV/PDF-2180 RBDs were below the detection limit (Supplementary Fig. S7a–d), while corresponding mutations significantly improved RBD binding efficiency, with apparent binding affinities (*K*_D,app_) ranging from 6.94 × 10^−10^ to 2.35 × 10^−8^ M (Fig. 4h, i). Restored receptor function of Rsin ACE2-N55T and Raeg ACE2-ABCD was also confirmed in a bat cell line Tb 1 Lu (Fig. 4j; Supplementary Fig. S7e, f). Together, our results demonstrated that unfavorable/sub-optional residues in the four predicted host range determinants restrict most Yin-bats’ ACE2 orthologues from effectively recognizing NeoCoV and PDF-2180.

### Genetic determinants restricting NeoCoV and PDF-2180 entry in non-bat mammals

**Fig. 5:**
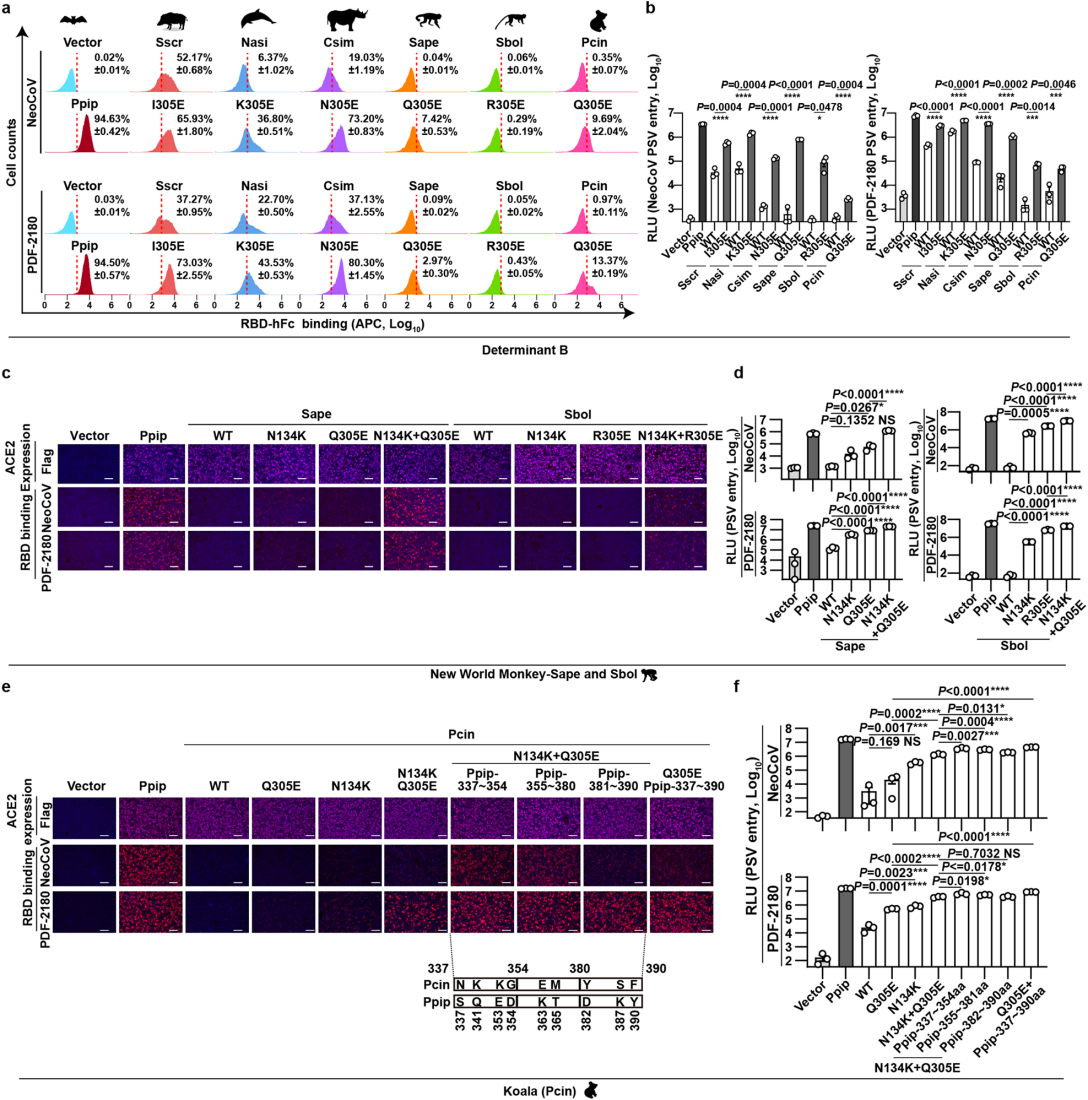
Determinants restricting NeoCoV and PDF-2180 recognition of ACE2 orthologues from non-bat mammals. **a**–**f** Gain of receptor function of indicated mammalian ACE2 orthologues in supporting NeoCoV and PDF-2180 RBD binding (**a**, **c**, **e**) and pseudoviruses entry (**b**, **d**, **f**) through the indicated mutations. **a** Flow cytometry analysis of NeoCoV and PDF-2180 RBD-hFc binding efficiencies with HEK293T cells transiently expressing the six non-supportive mammalian ACE2 and corresponding 305E mutants. The red dashed lines: the threshold for positive cells set by vector control. **a**, **b** 305E related mutants; **c**, **d** Sape and Sbol ACE2 related mutants; **e**, **f** Pcin ACE2 related mutants. Schematic illustrations of Pcin ACE2 mutants carrying substitutions of Ppip counterparts were shown in **e**. Data are presented as means ± SD for *n* = 3 biologically independent cells for **a**. Data are presented as means ± SEM for *n* = 3 biologically independent cells for **b**, **d**, and **f**. Data representative of two independent experiments for **b**–**f**. Two-tailed unpaired Student’s *t*-test; *P < 0.05, **P < 0.01, ***P < 0.005, and ****P < 0.001, NS not significant. RLU relative light unit. Scale bars represent 100 μm for **c** and **e**.

We next explored the genetic determinants restricting NeoCoV/PDF-2180 recognition of ACE2 orthologues from the six non-bat mammals: Pig (Sscr), Koala (Pcin), two closely related New World monkeys (Sape, Sbol), and two endangered animals Finless Porpoise (Nasi) and Southern white rhinoceroses (Csim). As predicted, none of the six deficient ACE2 orthologues possess an E at site 305, which is a crucial host range determinant that facilitates optimal salt bridge formation. Therefore, we generated 305E mutants for the six ACE2 orthologues and tested their ACE2 function. All these mutants were well-expressed, and the point mutation rendered efficient RBD binding for ACE2 orthologues from Sscr, Nasi, and Csim, but not for ACE2 orthologues from Sape, Sbol, and Pcin (Fig. 5a; Supplementary Fig. S8a). Aslo, the significant improvement of NeoCoV and PDF-2180 pseudovirus entry efficiency further confirmed the importance of E305 for better interaction (Fig. 5b).

Since the 305E mutation alone did not fully recover receptor function for Sape, Sbol, and Pcin ACE2, we further explored other genetic determinants restricting their recognition. For Sape and Sbol ACE2, we created chimeric ACE2 with specific regions substituted by the phylogenetically close-related Cjac ACE2. We observed a significant gain of receptor function in Sape when 1–251 aa or 125–251 aa were replaced by Cjac ACE2 counterparts. Fine mapping of region 125–251 aa targeted residue 134 as a specific genetic determinant for Sape and Sbol (Supplementary Fig. S8b–e). A better gain of receptor function of Sape and Sbol ACE2 orthologues can be observed upon N134K and Q/R305E double mutation (Fig. 5c, d; Supplementary Fig. S8f, g). Koala (Pcin) ACE2 is phylogenetically distant to ACE2 from other mammals (Supplementary Fig. S1b). A previous study reported that T31K and F83Y double mutations restored receptor function of Koala ACE2 to support SARS-CoV-2 entry^33^. However, the Pcin ACE2 with BCD substitutions remains defective in supporting NeoCoV and PDF-2180 binding and entry. We then generated Pcin ACE2 chimeras with specific regions replaced by Ppip ACE2 equivalent sequences (Supplementary Fig. S8h–k). Our result showed that a significant gain of entry-supporting ability could be achieved by mutant 2 (337–390 aa) and mutant 3 (280–390 aa), but not by mutant 1 (280–337 aa) (Supplementary Fig. S8l). Further mutation analysis indicated that the receptor function could be partially restored by Q305E + N134K, and further improved by additional substitution on 337–354 aa or 355–380 aa (Fig. 5e, f). Together, our results highlighted the critical role of site 305 for host tropism determination in mammals, despite the presence of other species-specific determinants beyond the interaction interface.

### RBM mutations further expand the potential host range of NeoCoV and PDF-2180

**Fig. 6:**
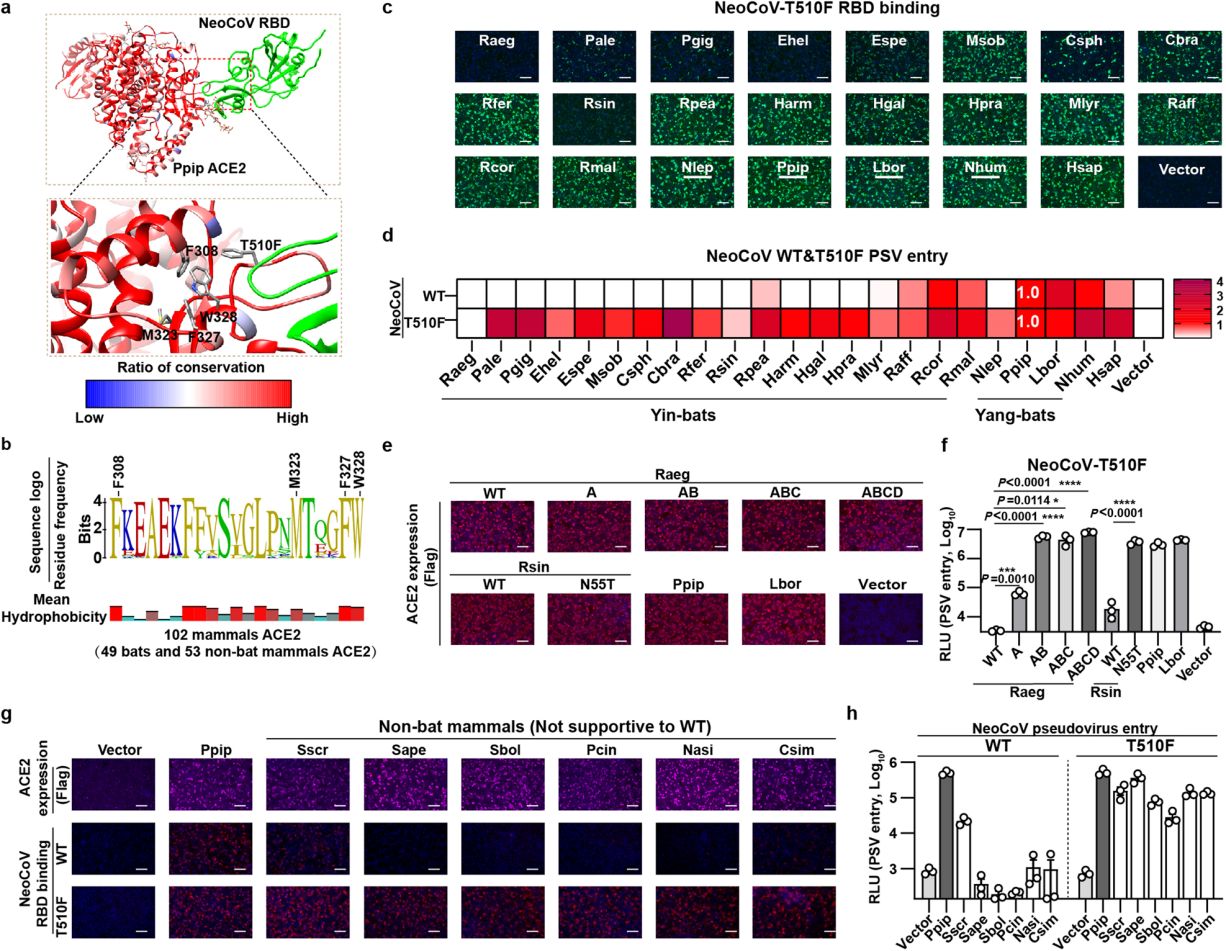
RBM mutation T510F further expands the potential host range of NeoCoV. **a** Structure showing the interaction interface between NeoCoV-T510F and a conserved hydrophobic pocket in Ppip ACE2. The side chains of hydrophobic residues constituting the pocket were indicated as sticks in the magnified view. The blue and red colors of the Ppip ACE2 represent the conservation ratio based on ACE2 orthologues from 49 bats and 53 non-bat mammals. **b** The sequence conservation of residues surrounding the conserved hydrophobic pocket based on ACE2 orthologues from 49 bats and 53 non-bat mammals. The residues that constitute the pocket were indicated with sequence numbers. The side chain hydrophobicity is shown below. **c**, **d** NeoCoV RBD-T510F-hFc RBD binding (**c**) and NeoCoV WT & T510F pseudoviruses entry (**d**) in HEK293T cells transiently expressing the indicated bat ACE2 orthologues. Entry efficiencies in PpipACE2 were set as 1.0. **e** The immunofluorescence analyzing the expression level of Raeg and Rsin ACE2 orthologues and their mutants. **f** The NeoCoV-T510F pseudovirus entry efficiency mediated by the indicated ACE2. **g**, **h** The efficiency of WT and NeoCoV-T510F RBD binding (**g**) and pseudovirus entry (**h**) in HEK293T cells transiently expressing the indicated mammalian ACE2 orthologues. Data are presented as mean for **d** and means ± SD for **f**, both are *n* = 3 biologically independent cells. Data are presented as means ± SEM for *n* = 3 biologically independent cells for **h**. Data representative of two independent experiments for **c**–**h**. Two-tailed unpaired Student’s *t*-test; *P < 0.05, **P < 0.01, ***P < 0.005, and ****P < 0.001. RLU relative light unit. Scale bars represent 100 μm for **c**, **e**, and **g**.

We previously showed that specific mutations in NeoCoV and PDF-2180 RBM confer more efficient hACE2 recognition^15^. Specifically, for NeoCoV, the substitution of its T510 by the PDF-2180 equivalent residue F511 (T510F) with higher hydrophobicity enhanced its interaction with a hydrophobic pocket of human ACE2 (Fig. 6a). For PDF-2180, a G to A mutation at site 510 (A509 in NeoCoV) together with four additional residue substitutions within 537–543 aa (PDF-2180-G510A+4Muts) improved its binding affinity with hACE2^15^. Sequence analysis of the 102 ACE2 orthologues tested in this study indicates residues constituting this hydrophobic pocket are highly conserved across mammals (Fig. 6b). Thus, we hypothesized that RBM mutations could expand the potential host range by enhancing hydrophobic interactions with this highly conserved pocket.

Our results show that NeoCoV-T510F efficiently binds with most tested ACE2 orthologues and can use these receptors for efficient entry, except for Rsin and Raeg ACE2 without the critical glycosylation in determinant A (Fig. 6c, d; Supplementary Fig. S4a). Further experiments demonstrated that Rsin ACE2-N55T and Raeg ACE2-I55T with functional N53 glycosylation sites also supported efficient NeoCoV-T510F entry (Fig. 6e, f). Additionally, NeoCoV-T510F also showed significantly improved ability to recognize the six non-bat mammalian ACE2 orthologues that are not recognized by the WT viruses (Fig. 6g, h). We observed similar results based on PDF-2180-G510A+4Muts, although the improvement is less prominent than the NeoCoV-T510F mutation (Supplementary Fig. S9a–e). These results indicate that NeoCoV, PDF-2180, or related viruses may extend their potential host range to Yin-bats and other non-permissive mammals, including humans, through antigenic drifts on RBM, such as T510F.

## Discussion

Global transmission of coronaviruses with higher pathogenicity, like MERS-CoV, has the potential to cause more devastating consequences than the COVID-19 pandemic^44^. Up to February 2023, MERS-CoV caused 2602 Laboratory-confirmed cases and 936 deaths worldwide since its emergence in Saudi Arabia in April 2012^2,11^. Fortunately, MERS-CoV appears to have relatively low transmissibility with a reproductive number (R0) around 0.69, which results in a gradual reduction of infected cases since 2016^11,45^. Whether this relatively low transmission rate is associated with the DPP4 receptor usage or the incomplete human adaptation remains an open question. However, the zoonotic emergence of MERS-CoV-related coronaviruses may occur and even develop into a large outbreak. The origin of MERS-CoV remains a mystery, although hypotheses have been proposed that MERS-CoV may arise from the recombination and evolution of MERS-related bat coronaviruses, such as HKU4, NeoCoV, and PDF-2180^12,46–49^.

Phylogenetically distant coronaviruses have evolved to employ ACE2 as their common receptors^19^. To date, coronaviruses of three different subgenera engage ACE2 for cellular entry, including NL63 (Setracovirus subgenus, α-CoV), many SARS-related CoVs (*Sarbecovirus* subgenus, β-CoV), and the two recently reported MERS-related CoVs (*Merbecovirus* subgenus, β-CoV) in this study. The distinct viral RBD structure and ACE2-binding footprints suggest convergent evolutionary histories of receptor acquisition and adaptation of these viruses. The reason for ACE2 preference among coronaviruses remains unknown. However, it is worth noting that ACE2 holds the potential to be used by coronaviruses to achieve efficient airborne transmission, considering the highly transmissible SARS-CoV-2 Omicron variant^50^. So far, structures similar to NeoCoV and PDF-2180 RBDs were not reported in other bat coronaviruses, and the closest RBD homologs were found in hedgehog merbecoviruses which do not recognize ACE2^15,22,39^. Thus, understanding the transmission ability and host tropism of NeoCoV and PDF-2180 is crucial in assessing the zoonotic risk of these viruses.

We have demonstrated that NeoCoV and PDF-2180 could efficiently use most ACE2 orthologues from 102 mammalian species across 11 orders, highlighting a potentially board host tropism of ACE2-using merbecoviruses. These viruses displayed a bat-specific phenotype preferring ACE2 orthologues from Yang-bats but not from most Yin-bats, which is not observed in NL63 and SARS-CoV-2. This bat ACE2 preference is in line with the observation that most merbecoviruses were sampled in bats belonging to the family *Vespertilionidae* (vesper bats)^5^, the largest family of Yangochiroptera (Supplementary Fig. S1a), including the hosts of NeoCoV and PDF-2180. In comparison, most sarbecoviruses were identified in Yin-bats, particularly *Rhinolophine* or *Hipposideros*^5,51^. The differences in host preference likely limited the opportunities for cross-lineage recombination of these high-risk viruses. Only 6 tested non-bat mammals from 5 different orders were found to be almost non-supportive, while ACE2 from humans and several other primates exhibited a relatively weak receptor function among the tested ACE2 orthologues.

We here revealed that specific residues in the virus-binding interface determine ACE2 tropism of merbecoviruses. Interestingly, glycan–protein interactions play a crucial role in ACE2 recognition of merbecoviruses, especially the crucial interaction mediated by N54-(or N53 in Rsin and Raeg) glycosylation (determinant A). Similar glycan–protein interaction in receptor engagement has also been reported in other coronaviruses, including SARS-CoV-2 and human-infecting CCoV-HuPn-2018^52,53^. It would be interesting to investigate their contribution to host tropism in other coronaviruses. Another glycan-related determinant is the N329 (or N330 in some species) glycosylation. As only some ACE2 orthologues from Yang-bats are glycosylated at this site, its contribution to binding affinity and species specificity is less prominent than the N54 glycosylation, probably due to the compensation of nearby protein–protein interactions, and it should be noted that all tested ACE2 orthologues from non-bat mammals carry the N54-glycosylation. In addition to glycosylation-related determinants, determinants B and D participate in protein–protein interactions required for effective receptor recognition, especially salt bridges. Although determinant D has been demonstrated to restrict human and bat ACE2 recognition, the interaction mediated by determinant B (E305) seems to play a vital role in host range determinants in both bats and non-bat mammals. Notably, there are variations in the importance and acceptability of different amino acids for each determinant. For example, E305 is optimal for salt bridge formation, N305 in some ACE2 orthologues may form sub-optional hydrogen bonds with the viruses, while K305 seems unable to interact with the viruses and may even result in steric hindrance. Beyond the virus-binding surface, additional determinants like N134K in ACE2 orthologues from the two New World primates (Sape and Sbol) and Koala (Pcin) also contribute to host tropism. The mechanism may involve their influence on the ACE2 structure that indirectly affects the viral binding. A full recovery of receptor function of the koala (Pcin) ACE2 can be achieved only through fragment substitution, suggesting multiple genetic determinants, beyond the critical sites 305 and 134, are restricting Koala from NeoCoV and PDF-2180 infection.

Although the incompatible receptor recognition sets a primary barrier for inter-species transmission of coronaviruses, viruses could achieve host jumping via adaptive antigenic drift^54^. Here we show that the T510F mutation in the NeoCoV spike, by increasing binding affinity via interacting with a conserved hydrophobic pocket, broadens the potential host range. Our results indicate that NeoCoV and related viruses hold the potential to break the current host range barrier via adaptive antigenic drift or recombination in bats or other mammals. It should be noted that host immune responses and other host factors required for viral infections also play important roles in receptor-independent host tropism determination^55^. Thus, it remains unknown whether NeoCoV carrying T510F mutant, which has not been found in nature, can readily infect humans. However, there might be more ACE2-using merbecoviruses with better human ACE2 recognition that remain out of our radar. Therefore, more attention should be paid to the surveillance of these viruses in the wild.

Together, we revealed a broad receptor usage of ACE2-using merbecoviruses across mammals and characterized the critical genetic determinants restricting the host range. Our study adds knowledge to the molecular basis of species-specific ACE2 recognition of merbecoviruses, highlighting the importance of in-depth research of these potentially high-risk viruses to prepare for potential future outbreaks.

## Materials and methods

### Cell lines

HEK293T (CRL-3216) and the bat epithelial cell line Tb 1 Lu (CCL-88) were purchased from American Type Culture Collection (ATCC). Cells were maintained by Dulbecco’s Modified Eagle Medium (DMEM, Monad, China) with 1% Penicillin/Streptomycin (P/S) and 10% Fetal Bovine Serum (FBS). An I1-Hybridoma (CRL-2700) cell line secreting a neutralizing mouse monoclonal antibody targeting the VSV glycoprotein (VSVG) was cultured in Minimum Essential Medium (MEM) with Earles’s balance salts and 2.0 mM of _L_-glutamine (Gibico) and 10% FBS. All the cells were cultured at 37 °C with 5% CO_2_ with regular passage every 2–3 days.

### Plasmids

Plasmids expressing WT or mutated bats ACE2 orthologues were generated by inserting human codon-optimized sequences with/without specific mutations into a lentiviral transfer vector (pLVX-EF1a-Puro, Genewiz) with C-terminus 3× Flag (DYKDHD-G-DYKDHD-I-DYKDDDDK). Human codon-optimized sequences of all non-bat mammalian ACE2 and their mutants were cloned into a vector (pLVX-IRES-zsGreen) with a C-terminal Flag tag (DYKDDDDK)^27^. Human codon-optimized spike sequences of NeoCoV (AGY29650.2), PDF-2180 (YP_009361857.1), SARS-CoV-2 (YP_009724390.1) carrying D614G mutation, and NL63 (APF29071.1) were cloned into the PCAGGS vector with C terminal deletions (13–15 aa) to improve the pseudovirus assembly efficiency. The plasmids expressing the recombinant CoVs RBD-hFc fusion proteins were constructed by inserting NeoCoV RBD (380–585 aa), PDF-2180 RBD (381-586 aa), SARS-CoV-2 RBD (331–524 aa) and NL63 RBD (481–616 aa) coding sequences into the pCAGGS vector containing an N-terminal CD5 secretion signal peptide (MPMGSLQPLATLYLLGMLVASVL) and a C-terminal hFc tag or hFc-twin-strep tandem tags for purification and detection. The plasmids expressing bats ACE2 ectodomain proteins were generated by inserting WT or mutated sequences of *Rousettus aegyptiacus* (18–740 aa), *Rhinolophus sinicus* (18–740 aa), *and Pipistrellus kuhlii* (20–738 aa) into the pCAGGS vector with an N-terminal CD5 secretion signal peptide and a C-terminal twin-strep-3×Flag tag (WSHPQFEKGGGSGGGSGGSAWSHPQFEKGGGRSDYKDHDGDYKDHDIDYKDDDDK). The species names, gene accession numbers, and ACE2 protein sequences were summarized in Supplementary Table S1.

### Protein expression and purification

HEK293T cells were transfected with different protein-expressing plasmids through the GeneTwin reagent (Biomed, TG101-01). At 4–6 h post-transfection, the medium of the transfected cells was replenished with the SMM 293-TII Expression Medium (Sino Biological, M293TII), and the protein-containing supernatant was harvested every 3 days for 2–3 batches. Recombinant RBD-hFc proteins were captured by Pierce Protein A/G Plus Agarose (Thermo Scientific, 20424), washed by wash buffer (100 mM Tris/HCl, pH 8.0, 150 mM NaCl, 1 mM EDTA), eluted with pH 3.0 Glycine buffer (100 mM in H_2_O), and then immediately balanced by 1/10 volume of UltraPure 1 M Tris-HCI, pH 8.0 (15568025, Thermo Scientific). Proteins with twin-strep tag were captured by Strep-Tactin XT 4Flow high-capacity resin (IBA, 2-5030-002), washed by wash buffer, and then eluted by buffer BXT (100 mM Tris/HCl, pH 8.0, 150 mM NaCl, 1 mM EDTA, 50 mM biotin). The eluted proteins were concentrated by Ultrafiltration tubes, buffer changed with PBS, and stored at –80 °C. Protein concentrations were determined by the Omni-Easy Instant BCA Protein Assay Kit (Epizyme, ZJ102).

### Coronavirus RBD-hFc live-cell binding assays

Different coronavirus RBD-hFc recombinant proteins were diluted in DMEM at indicated concentrations and incubated with HEK293T cells expressing different ACE2 for 30 min at 37 °C at 36 h post-transfection. After binding, cells were washed once by Hanks’ Balanced Salt Solution (HBSS) and then incubated with 1 μg/mL of Alexa Fluor 488-conjugated goat anti-human IgG (Thermo Fisher Scientific; A11013) or DyLight 594-conjugated goat anti-human IgG (Thermo Fisher Scientific; SA5-10136) diluted in PBS/1% BSA for 1 h at 37 °C. Next, cells were washed once by HBSS and then incubated with Hoechst 33342 (1:10,000 dilution in HBSS) for 30 min at 37 °C to stain the nucleus. Images were captured by a fluorescence microscope (MI52-N). The relative fluorescence units (RFU) of the stained cells were determined by a Varioskan LUX Multi-well Luminometer (Thermo Scientific). For flow cytometry analysis, HEK293T cells were transfected with the indicated ACE2 constructs and detached using 5 mM EDTA/PBS after 36 h. The cells were then washed twice with cold PBS and incubated with NeoCoV or PDF-2180 RBD-hFc-twin-strep (2 μg/mL) proteins at 4 °C for 30 min. ACE2-expressing cells were gated by zsGreen^+^ for non-bat mammalian ACE2 and Flag^+^ for bat ACE2, respectively. For non-bat mammalian ACE2, cells were washed twice with PBS and incubated with 1 μg/mL of anti-Strep-Tag II monoclonal antibody targeting the RBD (Abbkine; ABT2230) for 30 min at 4 °C. The cells were then washed twice with PBS and incubated with Alexa Fluor 647-conjugated goat anti-mouse IgG (Thermo Fisher Scientific; A32728) diluted in PBS/1% BSA for 1 h at 4 °C. For Bat ACE2, cells were incubated with Alexa Fluor 488-conjugated goat anti-human IgG for RBD staining (Thermo Fisher Scientific; A11013) at 4 °C for 1 h. The cells were fixed with 4% PFA, permeabilized with 0.25% Triton X-100, blocked with 1% BSA/PBS at 4 °C, and then stained with mouse antibody M2 (Sigma-Aldrich, F1804) diluted in PBS/1% BSA for 1 h at 4 °C to stain the ACE2 expression. After extensive washing, the cells were incubated with Alexa Fluor 647-conjugated goat anti-mouse IgG (Thermo Fisher Scientific; A32728) diluted in 1% BSA/PBS for 1 h at 4 °C. The stained cells were analyzed with a CytoFLEX Flow Cytometer (Beckman), and 10,000 events in a gated live cell population (based on SSC/FSC) were analyzed for all samples. HEK293T cells transfected with an empty vector plasmid were used as negative controls.

### BLI assays

BLI assays were performed on the Octet RED96 instrument (Molecular Devices) following the manufacturer’s instructions. In general, RBD-hFc recombinant proteins (20 μg/mL) were immobilized on the Protein A (ProA) biosensors (ForteBio, 18-5010) and incubated with 2-fold serial-diluted bat ACE2-ectodomain proteins starting from 500 nM in the kinetic buffer (PBST). The background was set with a kinetic buffer without the ACE2-ectodomain proteins. The kinetic parameters and binding affinities between the RBD-hFc and different bat ACE2 were analyzed by Octet Data Analysis software 12.2.0.20 through curve-fitting kinetic or steady-state analysis.

### Pseudovirus production and titration

VSV-dG-based pseudoviruses carrying coronavirus spike proteins were produced based on a modified protocol as previously described^56^. In general, HEK293T cells were transfected with CoV spike protein-expressing plasmids. At 24 h post-transfection, cells were transduced with 1.5 × 10^6^ TCID_50_ VSV-G glycoprotein-deficient VSV expressing GFP and firefly luciferase (VSV-dG-GFP-fLuc, generated in our lab) diluted in DMEM with 8 μg/mL polybrene for 4–6 h at 37 °C. After three times of PBS washes, the culture medium was replenished with DMEM + 10% FBS or SMM 293-TII Expression Medium (Sino Biological, M293TII) containing VSV neutralizing antibody (from I1-mouse hybridoma). 24 h later, the virus-containing supernatant was clarified through centrifugation at 4000 rpm for 5 min at 4 °C and then stored at –80 °C. TCID_50_ of pseudotyped viruses was determined based on three-fold serial dilution-based infection assays on HEK293T-bat40ACE2 cells for NeoCoV and PDF-2180 S pseudotypes, and 293T-hACE2 cells for NL63 and SARS-CoV-2 S pseudotypes. TCID_50_ was calculated according to the Reed-Muench method^57,58^.

### Pseudovirus entry assay

Pseudovirus entry assays were conducted on HEK293T or Tb 1 Lu cells transiently expressing WT or mutant ACE2 orthologues at 36 h post-transfection. In general, 3 × 10^4^ trypsinized cells were incubated with pseudovirus (1.5 × 10^5^ TCID_50_/100 μL) in a 96-well plate to allow attachment and viral entry. TPCK-trypsin (Sigma-Aldrich, T8802) treatment was conducted before NeoCoV and PDF-2180 pseudovirus entry assay. In this case, pseudovirus produced in Serum-free SMM 293-TII expression medium were incubated with 1–10 μg/mL (based on different levels of residual FBS from the I1-Hybridoma cultured medium and different batches of TPCK-treated trypsin) TPCK-treated trypsin for 10 mins at room temperature. The intracellular luciferase activity was measured by Bright-Glo Luciferase Assay Kit (Promega, E2620) and detected with a GloMax 20/20 Luminometer (Promega) at 18 h post-infection. GFP intensity was analyzed by a fluorescence microscope (Mshot, MI52-N).

### Western blot assay

For Western blot analysis, cells were lysed with 1% TritonX/PBS + 1 mM PMSF (Beyotime, ST506) for 10 min at 4 °C, then clarified through centrifugation of 12,000 rpm for 5 min at 4 °C. The clarified cell lysate was mixed with the 1/5 volume of 5× SDS loading buffer and incubated at 98 °C for 10 min. After gel electrophoresis and membrane transfer, the PVDF-membrane blots were blocked with 5% skimmed milk in PBST for 2 h at room temperature and then incubated 1 μg/mL anti-Flag mAb (Sigma, F1804), anti-glyceraldehyde-3-phosphate dehydrogenase (GAPDH) (AntGene, ANT325) PAb or anti-β-tubulin (Immmuno Way, YM3030) mAb diluted in PBST containing 1% milk overnight at 4 °C. After three times washing with PBST, the blots were incubated with Horseradish peroxidase (HRP)-conjugated secondary antibody AffiniPure Goat Anti-Mouse or Rabbit IgG (H + L) (Jackson Immuno Research, 115-035-003 or 111-035-003) in 1% skim milk in PBST and incubated for 1 h at room temperature. The blots were then washed three times by PBST and then visualized using an Omni-ECL Femto Light Chemiluminescence Kit (EpiZyme, SQ201) by a ChemiDoc MP Imaging System (Bio-Rad).

### Immunofluorescence assay

Immunofluorescence assays were conducted to verify the expression levels of ACE2 with C-terminal fused 3× Flag. In general, the transfected cells were incubated with 100% methanol for 10 min at room temperature for fixation and permeabilization. Cells were then incubated with a mouse antibody M2 (Sigma-Aldrich, F1804) diluted in PBS/1% BSA for 1 h at 37 °C, followed by extensive wash and the incubation of secondary antibody of Alexa Fluor 594-conjugated goat anti-mouse IgG (Thermo Fisher Scientific, A32742) diluted in 1% BSA/PBS for 1 h at 37 °C. Before visualization, the nucleus was stained blue with Hoechst 33342 reagent (1:5000 dilution in PBS). Images were captured and merged with a fluorescence microscope (Mshot, MI52-N).

### Bioinformatic and structural analysis

Sequence alignments of different bats’ ACE2 or non-bat mammalian ACE2 were performed either by the MUSCLE algorithm by MEGA-X (version 10.1.8) or ClustalW (https://www.genome.jp/tools-bin/clustalw) software. The residue usage frequency (sequence logo) and mean hydrophobicity of all ACE2 sequences were generated by the Geneious Prime software. Phylogenetic trees were produced using the maximal likelihood method in IQ-TREE (http://igtree.cibiv.univie.ac.at/) (1000 Bootstraps) and polished with iTOL (v6) (https://itol.embl.de/)^59^. The structures were shown by ChimeraX based on SARS-CoV-2 RBD & human ACE2 (PDB: 6M0J), NL63 RBD & human ACE2 (PDB: 3KBH), NeoCoV RBD & Ppip ACE2 (PDB: 7WPO) and PDF-2180 RBD & Ppip ACE2 (PDB: 7WPZ). RBD binding footprints and interaction details were analyzed and demonstrated using the UCSF ChimeraX^60^. Structural representatives of NeoCoV RBD interacting with WT or mutated Ppip ACE2 were analyzed using the UCSF ChimeraX. The structural representatives of NeoCoV RBD interacting with mutated Ppip ACE2 were reconstructed using Coot software (WinCoot version 0.9.4.1 EL)^61^. The indicated residues in PpipACE2 were changed using the mutate & autofitting map, and the structural representatives were refined using the sphere refine feature. Detailed illustrations of the interaction structure were extracted using UCSF ChimeraX. The colored sequence conservation of 102 mammals ACE2 was demonstrated by the UCSF Chimera based on multi-sequence alignments data generated by MEGA-X.

### Statistical analysis

Most experiments were conducted 2–3 times with 3 or 4 biological repeats. Representative results were shown. Data were presented by means ± SD or means ± SEM as indicated in the figure legends. Unpaired two-tailed *t*-tests were conducted for all statistical analyses using GraphPad Prism 8. *P* < 0.05 was considered significant. *P < 0.05, **P < 0.01, ***P < 0.005, and ****P < 0.001.

## Materials availability

All reagents generated in this study are available from the lead contact with a completed Materials Transfer Agreement.

## Supplementary information

Supplementary Information

Supplementary Table S1
