# Supplementary material for: Broad host tropism of ACE2-using MERS-related coronaviruses and determinants restricting viral recognition
Source: Cell Discov. 2023 Jun 15;9:57. doi: 10.1038/s41421-023-00566-8 (PMC10272122; doi:10.1038/s41421-023-00566-8)
Supplement: Supplementary file 1 — Supplementary Information [file 41421_2023_566_MOESM1_ESM.pdf]

Supplementary Information for

Broad host tropism of ACE2-using MERS-related coronaviruses and determinants restricting viral recognition

Chengbao Ma[1*], Chen Liu[1*], Qing Xiong[1*], Mengxue Gu[1], Lulu Shi[1], Chunli Wang[1], Junyu Si[1], Fei Tong[1], Peng Liu[1], Meiling Huang[1], Huan Yan[1] ✉

[1]State Key Laboratory of Virology, Institute for Vaccine Research and Modern Virology Research Center, College of Life Sciences, TaiKang Center for Life and Medical Sciences, Wuhan University, Wuhan, Hubei, China.

[*]These authors contributed equally.

✉Correspondence: huanyan@whu.edu.cn

This file includes:

Supplementary Figures S1 to S9

Gene information of ACE2 orthologues used in this study were summarized in: Supplementary Table S1

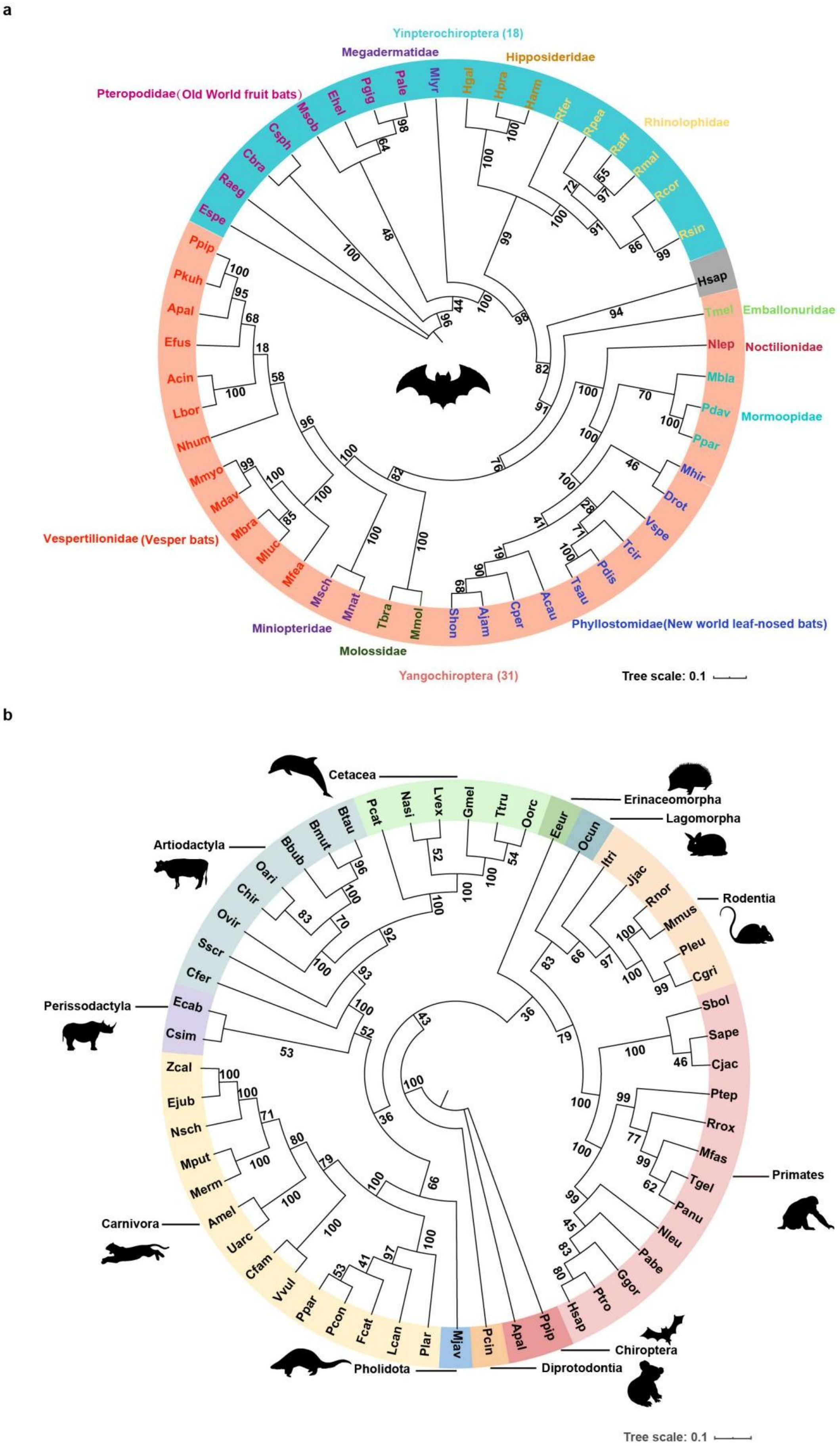
a
Yinpterochiroptera (18)
Megadermatidae
Hipposideridae
Pteropodidae（Old World fruit bats）
Rhinolophidae
Emballonuridae
Noctilionidae
Mormoopidae
Vespertilionidae (Vesper bats)
Miniopteridae
Molossidae
Phyllostomidae(New world leaf-nosed bats)
Yangochiroptera (31)
Tree scale: 0.1
Espe Raeg Cbra Csph Msob Ehel Pgig Pale Mlyr Hgal Hpra Harm Rfer Rpea Raff Rmal Rcor Rsin Hsap Tmel Nlep Mbla Pdav Ppar Mhir Drot Vspe Tcir Pdis Tsau Acau Cper Ajam Shon Mmol Tbra Mnat Msch Mfea Mluc Mbra Mdav Mmyo Nhum Lbor Acin Efus Apal Pkuh Ppip
b
Cetacea
Erinaceomorpha
Lagomorpha
Artiodactyla
Rodentia
Perissodactyla
Carnivora
Primates
Chiroptera
Pholidota
Diprotodontia
Tree scale: 0.1
Pcat Nasi Lvex Gmel Ttru Oorc Eeur Ocun Itri Jjac Rnor Mmus Pleu Cgri Sbol Sape Cjac Ptep Rrox Mfas Tgel Panu Nleu Pabe Ggor Ptro Hsap Ppip Apal Pcin Mjav Plar Lcan Fcat Pcon Ppar Vvul Cfam Uarc Amel Merm Mput Nsch Ejub Zcal Csim Ecab Cfer Sscr Ovir Chir Oari Bbub Bmut Btau

Supplementary Fig. S1 The phylogenetic trees of the bats and mammalian ACE2 tested in this study.

(**a, b**) The phylogenetic trees were generated by the IQ-TREE based on the ACE2 protein sequences from the 49 bats species (**a**) or 55 mammals (including two bats) (**b**)**.** Species of the same order or suborder were highlighted with different background colors. The GenBank accession numbers and protein sequences were summarized in **Supplementary Table S1**.

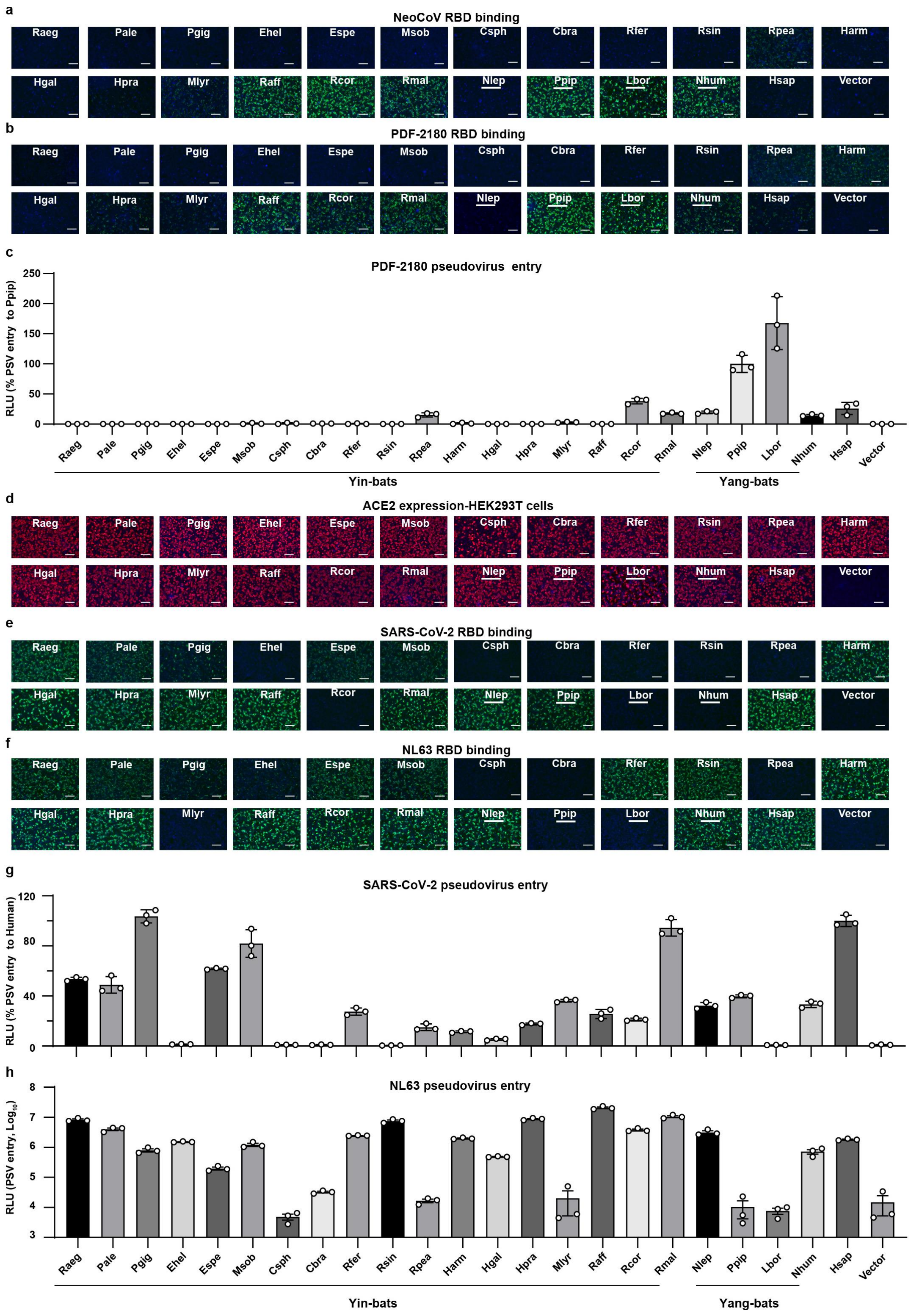
a
NeoCoV RBD binding
Raeg
Pale
Pgig
Ehel
Espe
Msob
Csph
Cbra
Rfer
Rsin
Rpea
Harm
Hgal
Hpra
Mlyr
Raff
Rcor
Rmal
Nlep
Ppip
Lbor
Nhum
Hsap
Vector
b
PDF-2180 RBD binding
c
PDF-2180 pseudovirus entry
RLU (% PSV entry to Ppip)
Yin-bats
Yang-bats
d
ACE2 expression-HEK293T cells
e
SARS-CoV-2 RBD binding
f
NL63 RBD binding
g
SARS-CoV-2 pseudovirus entry
RLU (% PSV entry to Human)
h
NL63 pseudovirus entry
RLU (PSV entry, Log10)

Supplementary Fig. S2 The RBD binding and pseudoviruses entry efficiency of ACE2-using CoVs in HEK293T cells transiently expressing the indicated bats ACE2 orthologues.

(**a-c**) The RBD binding (**a, b**) and pseudoviruses entry efficiency (**c**) of NeoCoV (**a**) and PDF-2180 (**b, c**) in HEK293T cells transiently expressing the indicated bats ACE2. (**d**) The expression level of ACE2 orthologues examined by immunofluorescence assay detecting the C terminal fused 3×Flag. (**e-h**) The RBD binding (**e, f**) and pseudoviruses entry efficiency (**g, h**) of SARS-CoV-2 (**e, g**) and NL63 (**f**, **h**) in HEK293T cells transiently expressing the indicated bats ACE2.

Data are presented as mean ± SD for n=3 biologically independent cells for **c** and **g.** Data are presented as mean ± SEM for n=3 biologically independent cells for **h**. Data representative of two independent experiments. RLU: relative luciferase unit. Scale bar represents 100 μm for **a**, **b**, **d, e,** and **f.**

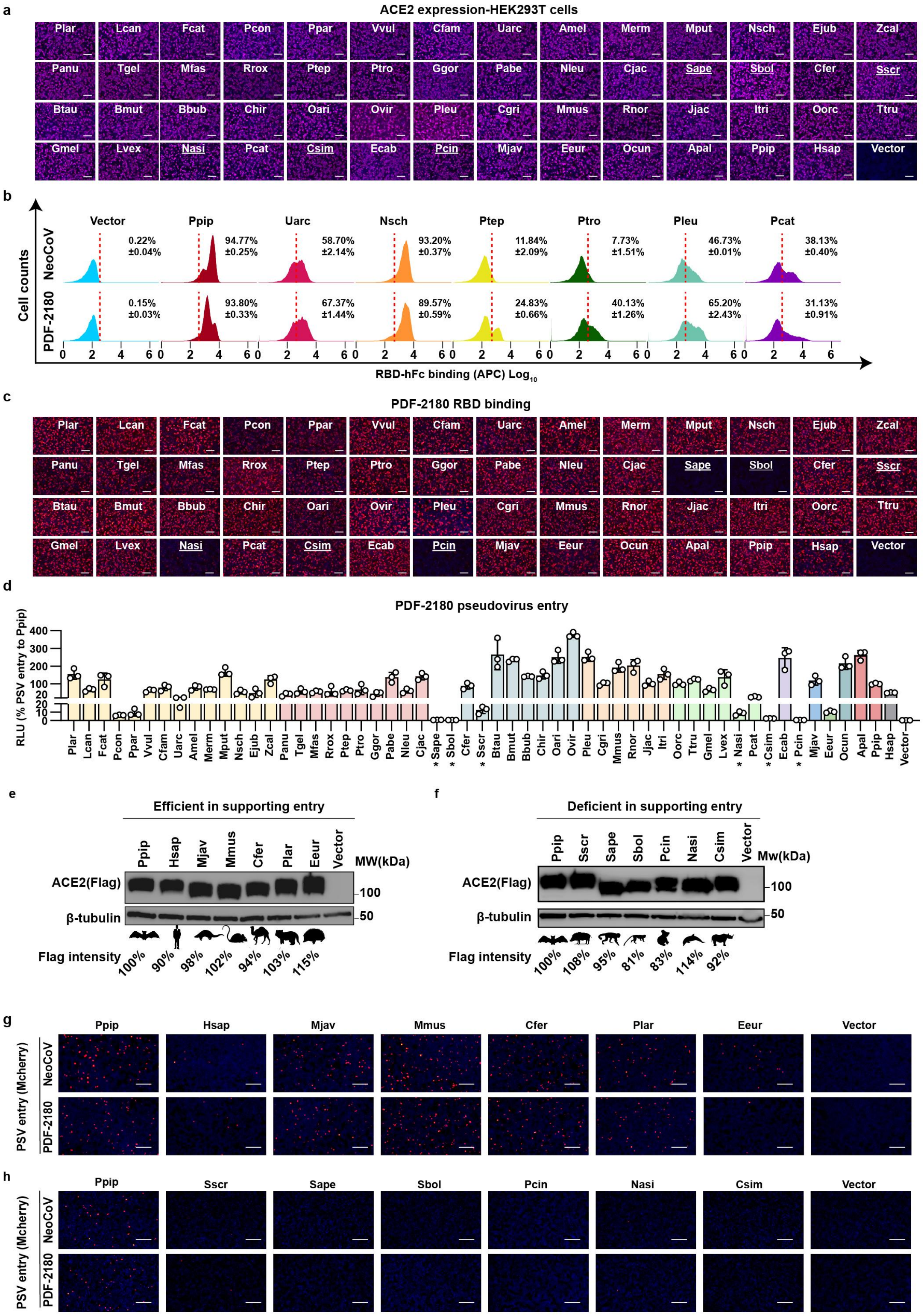
a
ACE2 expression-HEK293T cells
Plar Lcan Fcat Pcon Ppar Vvul Cfam Uarc Amel Merm Mput Nsch Ejub Zcal
Panu Tgel Mfas Rrox Ptep Ptro Ggor Pabe Nleu Cjac Sape Sbol Cfer Sscr
Btau Bmut Bbub Chir Oari Ovir Pleu Cgri Mmus Rnor Jjac Itri Oorc Ttru
Gmel Lvex Nasi Pcat Csim Ecab Pcin Mjav Eeur Ocun Apal Ppip Hsap Vector
b
Cell counts
NeoCoV
PDF-2180
Vector Ppip Uarc Nsch Ptep Ptro Pleu Pcat
0.22% ±0.04%
94.77% ±0.25%
58.70% ±2.14%
93.20% ±0.37%
11.84% ±2.09%
7.73% ±1.51%
46.73% ±0.01%
38.13% ±0.40%
0.15% ±0.03%
93.80% ±0.33%
67.37% ±1.44%
89.57% ±0.59%
24.83% ±0.66%
40.13% ±1.26%
65.20% ±2.43%
31.13% ±0.91%
RBD-hFc binding (APC) Log10
c
PDF-2180 RBD binding
d
PDF-2180 pseudovirus entry
RLU (% PSV entry to Ppip)
e
Efficient in supporting entry
Ppip Hsap Mjav Mmus Cfer Plar Eeur Vector
MW(kDa)
ACE2(Flag)
β-tubulin
100
50
Flag intensity
100% 90% 98% 102% 94% 103% 115%
f
Deficient in supporting entry
Ppip Sscr Sape Sbol Pcin Nasi Csim Vector
Mw(kDa)
ACE2(Flag)
β-tubulin
100
50
Flag intensity
100% 108% 95% 81% 83% 114% 92%
g
PSV entry (Mcherry)
NeoCoV
PDF-2180
Ppip Hsap Mjav Mmus Cfer Plar Eeur Vector
h
PSV entry (Mcherry)
NeoCoV
PDF-2180
Ppip Sscr Sape Sbol Pcin Nasi Csim Vector

Supplementary Fig. S3 NeoCoV and PDF-2180 can recognize most ACE2 orthologues from non-bat mammals.

(**a**) The expression level of ACE2 orthologues in HEK293T cells as indicated by immunofluorescence assay detecting the C-terminal Flag tag. (**b**) Flow cytometry analysis of NeoCoV and PDF-2180 RBD-hFc binding with HEK293T cells transiently expressing indicated ACE2 orthologues. Species with inconsistent results between RBD binding and PSV infection. The vector was used as a negative control. The red dashed lines indicate the threshold to define positive cells. (**c**) Entry efficiency of PDF-2180 pseudovirus in HEK293T cells transiently expressing mammalian ACE2. RLU %: PSV entry to Ppip. *: RLU<20% RLU_{Ppip} of NeoCoV pseudovirus entry. Species belonging to different orders were indicated with different background colors. (**d**) PDF-2180 RBD-hFc binding efficiency in HEK293T cells expressing indicated ACE2 from non-bat mammals as indicated by immunofluorescence assay detecting the hFc. (**e, f**) Western blot showing the expression of ACE2 orthologues from selected CoV host-related species (**e**) or NeoCoV/PDF-2180 non-supportive species (**f**) in HEK293T cells. (**g, h**) Entry efficiency of NeoCoV and PDF-2180 pseudoviruses (mcherry) in HEK293T cells transiently expressing indicated ACE2 orthologues from CoV host-related species (**g**) or non-permissive species (**h**).

Data are presented as mean ± SD for n=3 biologically independent cells. Data representative of two independent experiments. RLU: relative luciferase unit. Scale bar represents 100 μm for **a** and **d,** and 200 μm for **g** and **h**. Mw: molecular weight.

a

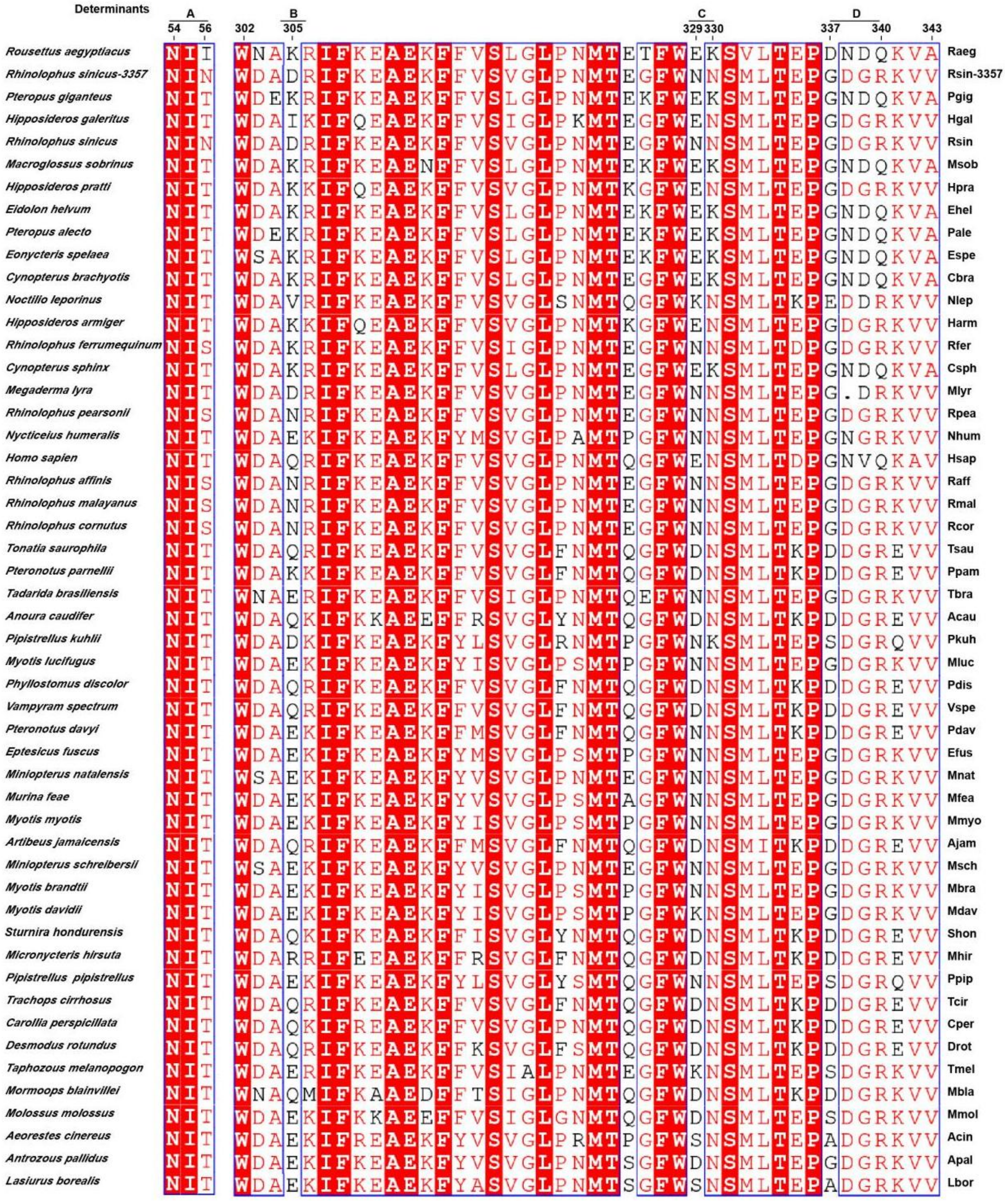
Determinants
A
54 56
B
302 305
C
329 330
D
337 340 343
Rousettus aegyptiacus Raeg
Rhinolophus sinicus-3357 Rsin-3357
Pteropus giganteus Pgig
Hipposideros galeritus Hgal
Rhinolophus sinicus Rsin
Macroglossus sobrinus Msob
Hipposideros pratti Hpra
Eidolon helvum Ehel
Pteropus alecto Pale
Eonycteris spelaea Espe
Cynopterus brachyotis Cbra
Noctilio leporinus Nlep
Hipposideros armiger Harm
Rhinolophus ferrumequinum Rfer
Cynopterus sphinx Csph
Megaderma lyra Mlyr
Rhinolophus pearsonii Rpea
Nycticeius humeralis Nhum
Homo sapien Hsap
Rhinolophus affinis Raff
Rhinolophus malayanus Rmal
Rhinolophus cornutus Rcor
Tonatia saurophila Tsau
Pteronotus parnellii Ppam
Tadarida brasiliensis Tbra
Anoura caudifer Acau
Pipistrellus kuhlii Pkuh
Myotis lucifugus Mluc
Phyllostomus discolor Pdis
Vampyram spectrum Vspe
Pteronotus davyi Pdav
Eptesicus fuscus Efus
Miniopterus natalensis Mnat
Murina feae Mfea
Myotis myotis Mmyo
Artibeus jamaicensis Ajam
Miniopterus schreibersii Msch
Myotis brandtii Mbra
Myotis davidii Mdav
Sturnira hondurensis Shon
Micronycteris hirsuta Mhir
Pipistrellus pipistrellus Ppip
Trachops cirrhosus Tcir
Carollia perspicillata Cper
Desmodus rotundus Drot
Taphozous melanopogon Tmel
Mormoops blainvillei Mbla
Molossus molossus Mmol
Aeorestes cinereus Acin
Antrozous pallidus Apal
Lasiurus borealis Lbor

Supplementary Fig. S4 The multi-sequence alignments of viral binding loops of bat ACE2 orthologues.

(a) The sequence alignment analysis was conducted based on viral binding loops of ACE2 by the Clustal W and rendered with ESPript. Identical residues are highlighted with a red background, and similar residues are colored red in blue boxes. The amino acid numbers correspond to the Ppip ACE2 number are indicated on the top.

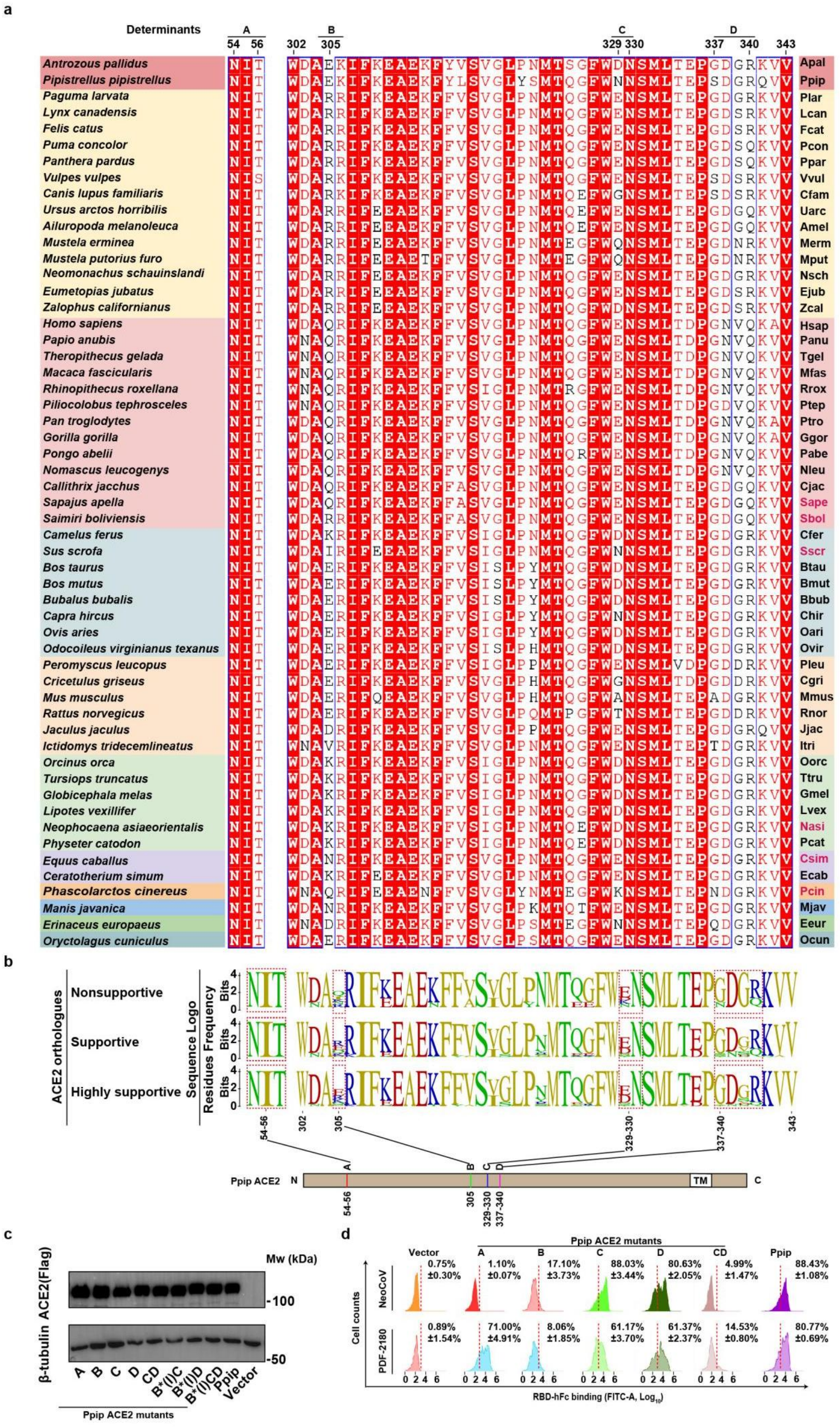
a
Determinants
A
B
C
D
54
56
302
305
329
330
337
340
343
Antrozous pallidus
Pipistrellus pipistrellus
Paguma larvata
Lynx canadensis
Felis catus
Puma concolor
Panthera pardus
Vulpes vulpes
Canis lupus familiaris
Ursus arctos horribilis
Ailuropoda melanoleuca
Mustela erminea
Mustela putorius furo
Neomonachus schauinslandi
Eumetopias jubatus
Zalophus californianus
Homo sapiens
Papio anubis
Theropithecus gelada
Macaca fascicularis
Rhinopithecus roxellana
Piliocolobus tephrosceles
Pan troglodytes
Gorilla gorilla
Pongo abelii
Nomascus leucogenys
Callithrix jacchus
Sapajus apella
Saimiri boliviensis
Camelus ferus
Sus scrofa
Bos taurus
Bos mutus
Bubalus bubalis
Capra hircus
Ovis aries
Odocoileus virginianus texanus
Peromyscus leucopus
Cricetulus griseus
Mus musculus
Rattus norvegicus
Jaculus jaculus
Ictidomys tridecemlineatus
Orcinus orca
Tursiops truncatus
Globicephala melas
Lipotes vexillifer
Neophocaena asiaeorientalis
Physeter catodon
Equus caballus
Ceratotherium simum
Phascolarctos cinereus
Manis javanica
Erinaceus europaeus
Oryctolagus cuniculus
Apal
Ppip
Plar
Lcan
Fcat
Pcon
Ppar
Vvul
Cfam
Uarc
Amel
Merm
Mput
Nsch
Ejub
Zcal
Hsap
Panu
Tgel
Mfas
Rrox
Ptep
Ptro
Ggor
Pabe
Nleu
Cjac
Sape
Sbol
Cfer
Sscr
Btau
Bmut
Bbub
Chir
Oari
Ovir
Pleu
Cgri
Mmus
Rnor
Jjac
Itri
Oorc
Ttru
Gmel
Lvex
Nasi
Pcat
Csim
Ecab
Pcin
Mjav
Eeur
Ocun
b
ACE2 orthologues
Nonsupportive
Supportive
Highly supportive
Sequence Logo
Residues Frequency
Bits
54-56
302
305
329-330
337-340
343
Ppip ACE2
N
C
TM
c
β-tubulin
ACE2(Flag)
Mw (kDa)
-100
-50
A
B
C
D
CD
B*(I)C
B*(I)D
B*(I)CD
Ppip
Vector
Ppip ACE2 mutants
d
Ppip ACE2 mutants
Vector
A
B
C
D
CD
Ppip
NeoCoV
PDF-2180
Cell counts
0.75% ±0.30%
1.10% ±0.07%
17.10% ±3.73%
88.03% ±3.44%
80.63% ±2.05%
4.99% ±1.47%
88.43% ±1.08%
0.89% ±1.54%
71.00% ±4.91%
8.06% ±1.85%
61.17% ±3.70%
61.37% ±2.37%
14.53% ±0.80%
80.77% ±0.69%
0 2 4 6
RBD-hFc binding (FITC-A, Log10)

Supplementary Fig. S5 The multi-sequence alignments and sequence conservation analysis of viral binding loops of ACE2 orthologues from 55 mammals.

(**a**) Multi-sequence alignment based on viral binding loops from 55 mammals (including two bats) by the Clustal W and rendered with ESPript. Identical residues were highlighted in red, and similar residues were in the blue frames. The amino acid numbers correspond to the Ppip ACE2 number are indicated on the top. (**b**) Sequence conservation analysis of the ACE2 orthologues grouped by their ability to support NeoCoV entry. Upper: non-supportive (<20% RLU_{Ppip}, 6 species); middle: supportive (> 20% RLU_{Ppip}, 49 species); lower: highly supportive (>100% RLU_{Ppip}, 30 species). (**c**) Western blot analysis of the WT and mutated Ppip ACE2 expression in HEK293T cells. (**d**) Flow cytometry analysis of NeoCoV and PDF-2180 RBD-hFc binding efficiencies with HEK293T cells transiently expressing the indicated Ppip WT and mutated ACE2 orthologues. The red dashed lines indicate the threshold to define positive cells. Data representative of two independent experiments for **c** and **d**. Mw: molecular weight.

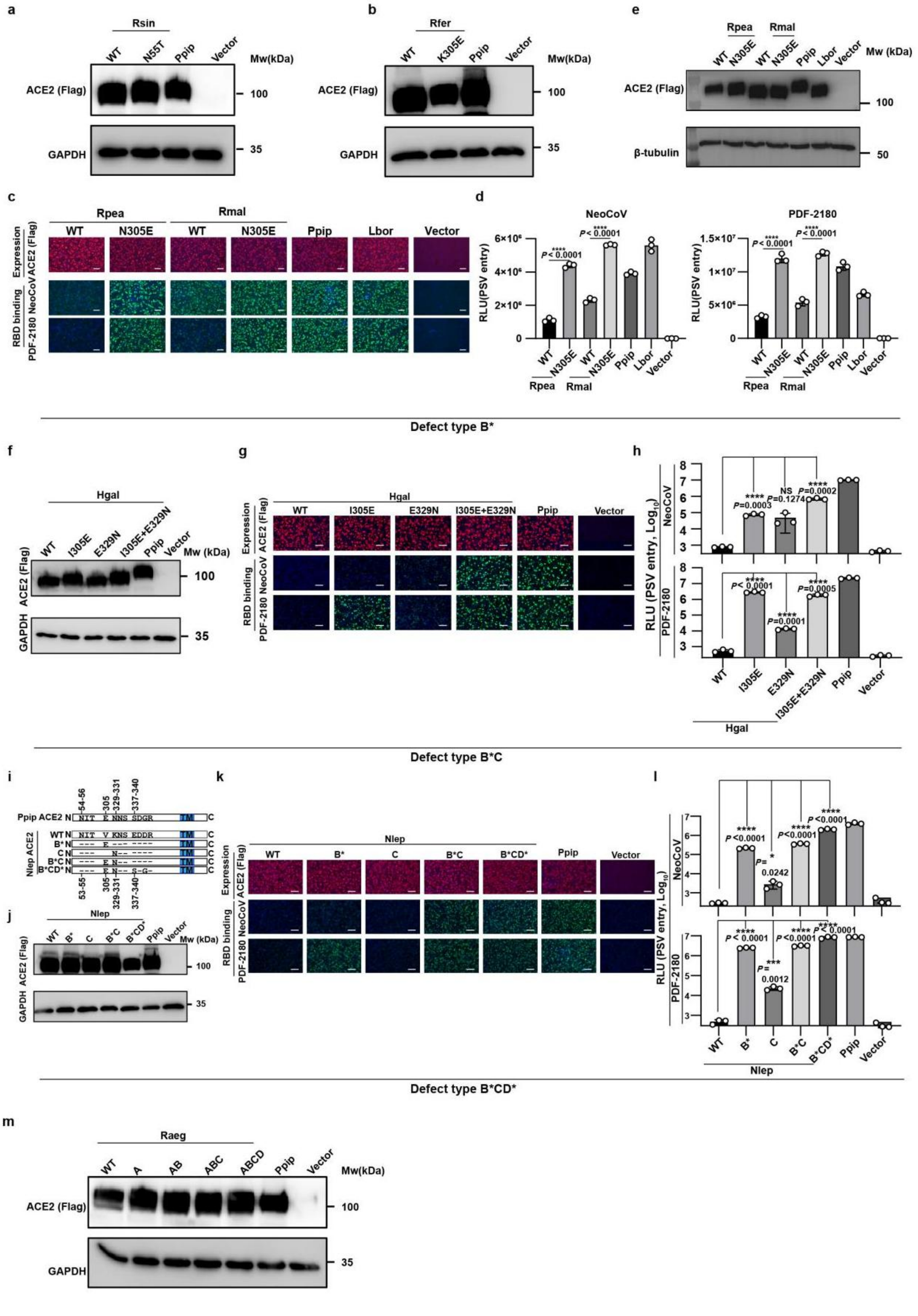
a
Rsin
WT
N55T
Ppip
Vector
Mw(kDa)
ACE2 (Flag)
100
GAPDH
35
b
Rfer
WT
K305E
Ppip
Vector
Mw(kDa)
ACE2 (Flag)
100
GAPDH
35
e
Rpea
Rmal
WT
N305E
WT
N305E
Ppip
Lbor
Vector
Mw (kDa)
ACE2 (Flag)
100
β-tubulin
50
c
Rpea
Rmal
WT
N305E
WT
N305E
Ppip
Lbor
Vector
Expression ACE2 (Flag)
RBD binding NeoCoV
PDF-2180
d
NeoCoV
PDF-2180
RLU(PSV entry)
P < 0.0001
Rpea
Rmal
Defect type B*
f
Hgal
WT
I305E
E329N
I305E+E329N
Ppip
Vector
Mw (kDa)
ACE2 (Flag)
100
GAPDH
35
g
Hgal
WT
I305E
E329N
I305E+E329N
Ppip
Vector
h
RLU (PSV entry, Log10)
NeoCoV
PDF-2180
P=0.0003
NS P=0.1274
P=0.0002
P< 0.0001
P=0.0001
P=0.0005
Hgal
Defect type B*C
i
Ppip ACE2
Nlep ACE2
WT
B*
C
B*C
B*CD*
TM
k
Nlep
WT
B*
C
B*C
B*CD*
Ppip
Vector
Expression ACE2 (Flag)
RBD binding NeoCoV
PDF-2180
j
Nlep
Mw (kDa)
ACE2 (Flag)
100
GAPDH
35
l
NeoCoV
PDF-2180
RLU (PSV entry, Log10)
P=0.0242
P=0.0012
P< 0.0001
Nlep
Defect type B*CD*
m
Raeg
WT
A
AB
ABC
ABCD
Ppip
Vector
Mw(kDa)
ACE2 (Flag)
100
GAPDH
35

Supplementary Fig. S6 Verification of bats ACE2 mutants with improved functionality to support NeoCoV/PDF-2180 RBD binding and pseudoviruses entry.

(**a, b**) Western blot analysis of the expression level of WT and mutated Rsin (**a**), Rfer (**b**) ACE2 in HEK293T cells. (**c**) Expression level and NeoCoV/PDF-2180 RBD-hFc binding efficiency of the WT and mutated Rpea and Rmal ACE2 by immunofluorescence in HEK293T cells. (**d**) The NeoCoV and PDF-2180 pseudoviruses entry efficiency supported by WT and mutated Rpea and Rmal ACE2. **(e)** Western blot analysis of the WT and mutated Rpea or Rmal ACE2 expression in HEK293T cells. (**f**) Western blot analysis of the WT and mutated Hgal ACE2 expression in HEK293T cells. (**g**) Expression levels and RBD binding supporting ability of WT and mutated Hgal ACE2. (**h**) The NeoCoV and PDF-2180 pseudoviruses entry efficiency supported by WT and mutated Hgal ACE2. (**i**) Schematic illustration of Nlep ACE2 swap mutants carrying the indicated PpipACE2 counterparts. (**j**) Western blot analysis of the expression level of WT and mutated Nlep ACE2 in HEK293T cells. (**k**) The binding efficiency of NeoCoV and PDF-2180 RBD supported by WT and mutated Nlep ACE2. (**l**) Entry efficiency of NeoCoV and PDF-2180 pseudoviruses supported by WT and mutated Nlep ACE2. (**m**) Western blot analysis of the expression levels of WT and mutated Raeg ACE2 in HEK293T cells.

Data are presented as mean ± SD for n=3 biologically independent cells for **d**, **h**, **l**. Data representative of three independent experiments for **c-h** and **j-l.** Data representative of two experiments for **a, b,** and **m**. Two-tailed unpaired Student's t-test; * P<0.05, ** P <0.01, *** P <0.005, and **** P <0.001. NS: not significant. RLU: relative luciferase unit. Mw: molecular weight. Scale bar represents 100 μm for **c**, **g,** and **k**.

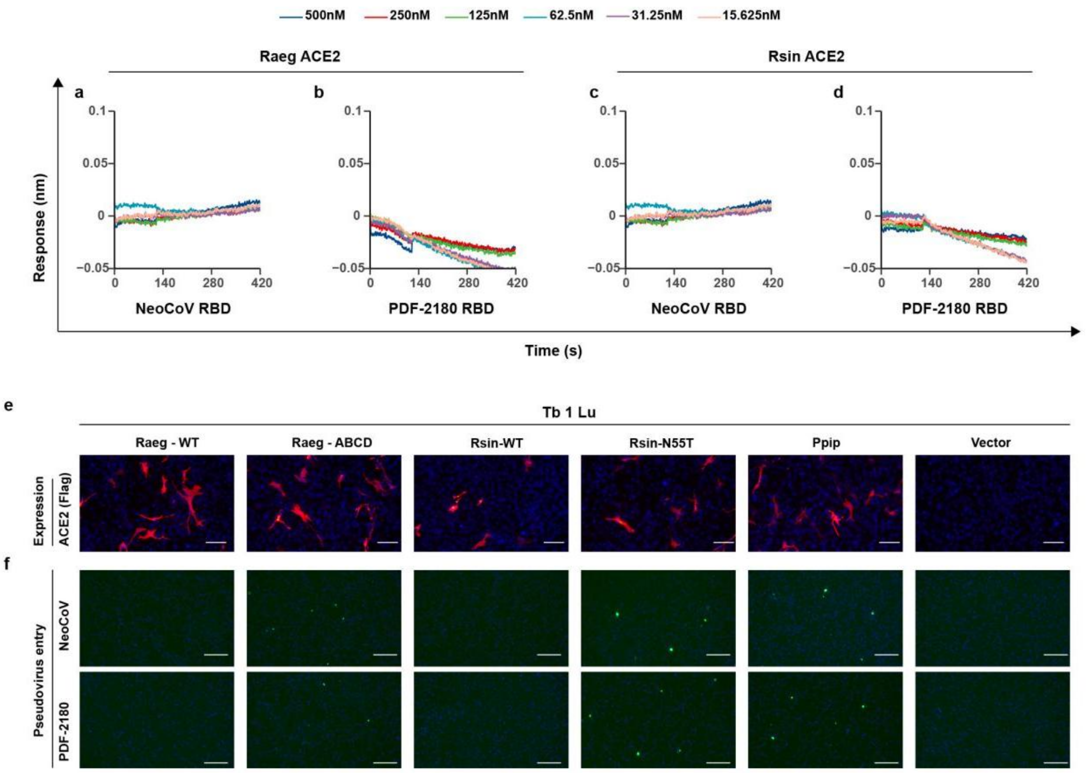
500nM
250nM
125nM
62.5nM
31.25nM
15.625nM
Raeg ACE2
Rsin ACE2
a
b
c
d
Response (nm)
0.1
0.05
0
−0.05
0
140
280
420
NeoCoV RBD
PDF-2180 RBD
Time (s)
e
f
Tb 1 Lu
Raeg - WT
Raeg - ABCD
Rsin-WT
Rsin-N55T
Ppip
Vector
Expression
ACE2 (Flag)
Pseudovirus entry
NeoCoV
PDF-2180

Supplementary Fig. S7 Verification of the receptor function of Raeg and Rsin ACE2 mutants with improved receptor recognition.

(**a-d**) BLI assays analyzing the binding kinetics between NeoCoV-RBD-hFc/PDF-2180-RBD-hFc and WT Raeg and Rsin ACE2 ectodomain proteins. (**e, f**) Verification of the gain of receptor function of Raeg and Rsin ACE2 mutants in Tb 1 Lu bat cell line. Immunofluorescence analysis of ACE2 expression level of Ppip and mutated Raeg and Rsin ACE2 in Tb1 Lu cells (**e**). NeoCoV and PDF-2180 pseudoviruses entry efficiency at 16 h post-infection as indicated by the GFP intensity (**f**).

Data representative of two independent experiments. Scale bar represents 100 μm for **e** and 200μm for **f.**

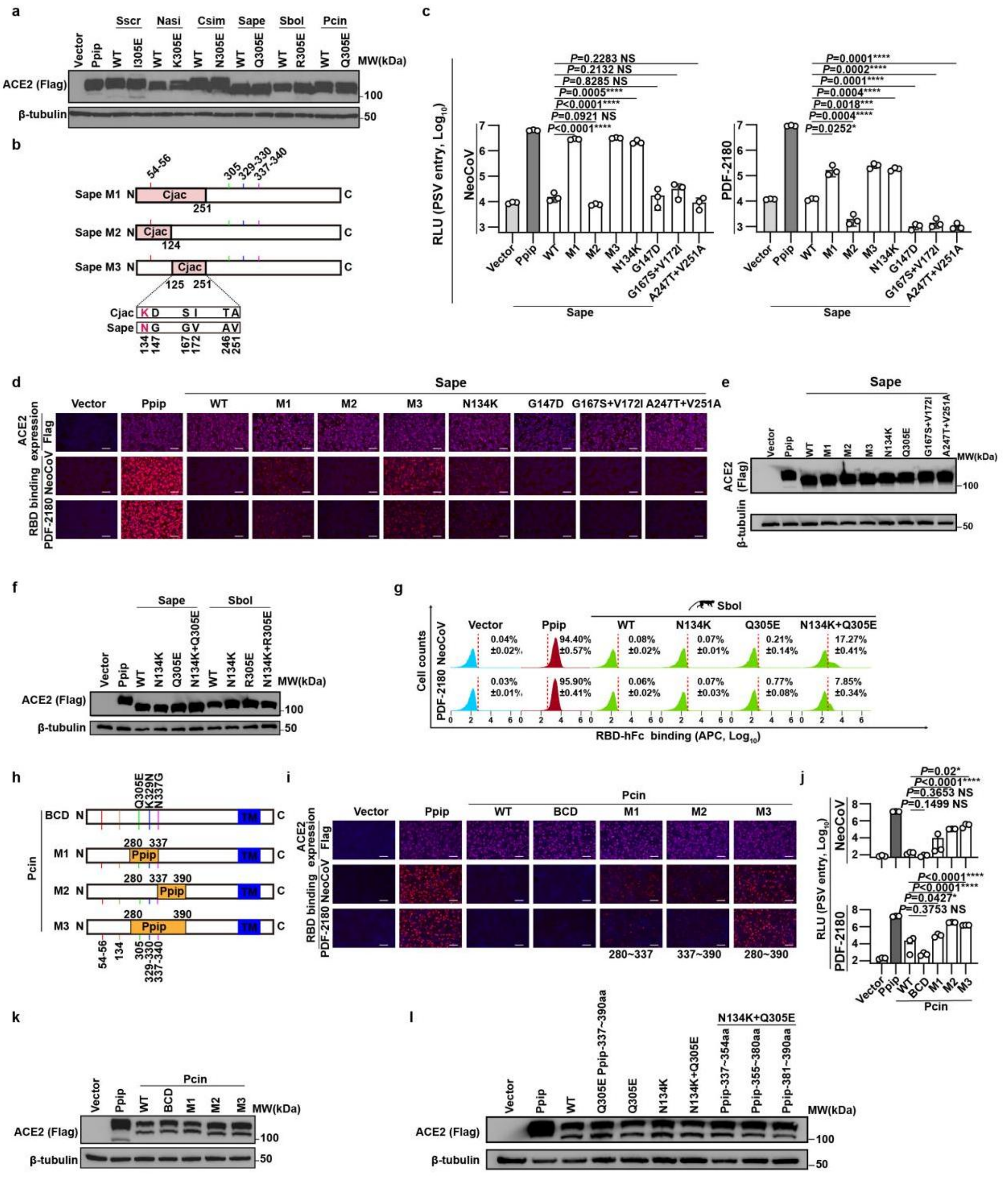

a
Vector
Ppip
Sscr
WT
I305E
Nasi
WT
K305E
Csim
WT
N305E
Sape
WT
Q305E
Sbol
WT
R305E
Pcin
WT
Q305E
MW(kDa)
ACE2 (Flag)
100
β-tubulin
50
b
54-56
305
329-330
337-340
Sape M1
Cjac
251
Sape M2
Cjac
124
Sape M3
Cjac
125
251
Cjac K D S I T A
Sape N G G V A V
134
147
167
172
246
251
c
RLU (PSV entry, Log10)
NeoCoV
P=0.2283 NS
P=0.2132 NS
P=0.8285 NS
P=0.0005****
P<0.0001****
P=0.0921 NS
P<0.0001****
PDF-2180
P=0.0001****
P=0.0002****
P=0.0001****
P=0.0004****
P=0.0018***
P=0.0004****
P=0.0252*
Vector
Ppip
WT
M1
M2
M3
N134K
G147D
G167S+V172I
A247T+V251A
Sape
d
Vector
Ppip
Sape
WT
M1
M2
M3
N134K
G147D
G167S+V172I
A247T+V251A
ACE2 expression
Flag
RBD binding
NeoCoV
PDF-2180
e
Sape
Vector
Ppip
WT
M1
M2
M3
N134K
Q305E
G167S+V172I
A247T+V251A
MW(kDa)
ACE2 (Flag)
100
β-tubulin
50
f
Sape
Sbol
Vector
Ppip
WT
N134K
Q305E
N134K+Q305E
WT
N134K
R305E
N134K+R305E
MW(kDa)
ACE2 (Flag)
100
β-tubulin
50
g
Sbol
Vector
Ppip
WT
N134K
Q305E
N134K+Q305E
Cell counts
NeoCoV
0.04% ±0.02%
94.40% ±0.57%
0.08% ±0.02%
0.07% ±0.01%
0.21% ±0.14%
17.27% ±0.41%
PDF-2180
0.03% ±0.01%
95.90% ±0.41%
0.06% ±0.02%
0.07% ±0.03%
0.77% ±0.08%
7.85% ±0.34%
RBD-hFc binding (APC, Log10)
h
Q305E
K329N
N337G
BCD
TM
Pcin
M1
280
337
Ppip
M2
280
337
390
M3
280
390
54-56
134
305
329-330
337-340
i
Vector
Ppip
Pcin
WT
BCD
M1
M2
M3
ACE2 expression
Flag
RBD binding
NeoCoV
PDF-2180
280~337
337~390
280~390
j
P=0.02*
P<0.0001****
P=0.3653 NS
P=0.1499 NS
NeoCoV
RLU (PSV entry, Log10)
P<0.0001****
P<0.0001****
P=0.0427*
P=0.3753 NS
PDF-2180
Vector
Ppip
WT
BCD
M1
M2
M3
Pcin
k
Pcin
Vector
Ppip
WT
BCD
M1
M2
M3
MW(kDa)
ACE2 (Flag)
100
β-tubulin
50
l
N134K+Q305E
Vector
Ppip
WT
Q305E Ppip-337~390aa
Q305E
N134K
N134K+Q305E
Ppip-337~354aa
Ppip-355~380aa
Ppip-381~390aa
MW(kDa)
ACE2 (Flag)
100
β-tubulin
50

Supplementary Fig. S8 Critical determinants restricting Sape and Pcin ACE2 orthologues from supporting NeoCoV and PDF-2180 RBD binding and pseudoviruses entry.

(**a**) Western blot analyzing the expression level of ACE2 orthologues mentioned in **Fig. 5a, b**. (**b**) Schematic illustration of Sape ACE2 swap mutants carrying the indicated Cjac ACE2 counterparts. (**c**-**e**) Identification of the critical determinants restricting Sape ACE2 from supporting NeoCoV/PDF-2180 pseudoviruses entry (**c**) and RBD binding (**e**) in HEK293T cells. (**d**) Western blot showing the expression of the WT and mutated Sape ACE2 orthologues. (**f**) Western blot analyzing the expression level of ACE2 orthologues mentioned in **Fig. 5c, d**. (**g**) Flow cytometry analysis of NeoCoV and PDF-2180 RBD-hFc binding with HEK293T cells transie **(k)** Western blot showing the expression of the WT and mutated Pcin ACE2. (**l**) Western blot showing the expression of the WT and mutated Pcin ACE2 orthologues mentioned in **Fig. 5e, f.**

Data are presented as mean ± SEM for n=3 biologically independent cells for **c** and **j**. Data representative of two independent experiments. Two-tailed unpaired Student's t-test; * P<0.05, ** P<0.01, ***P<0.005, and **** P<0.001. NS: not significant. RLU: relative luciferase unit. Scale bar represents 100 μm for **d** and **i**.

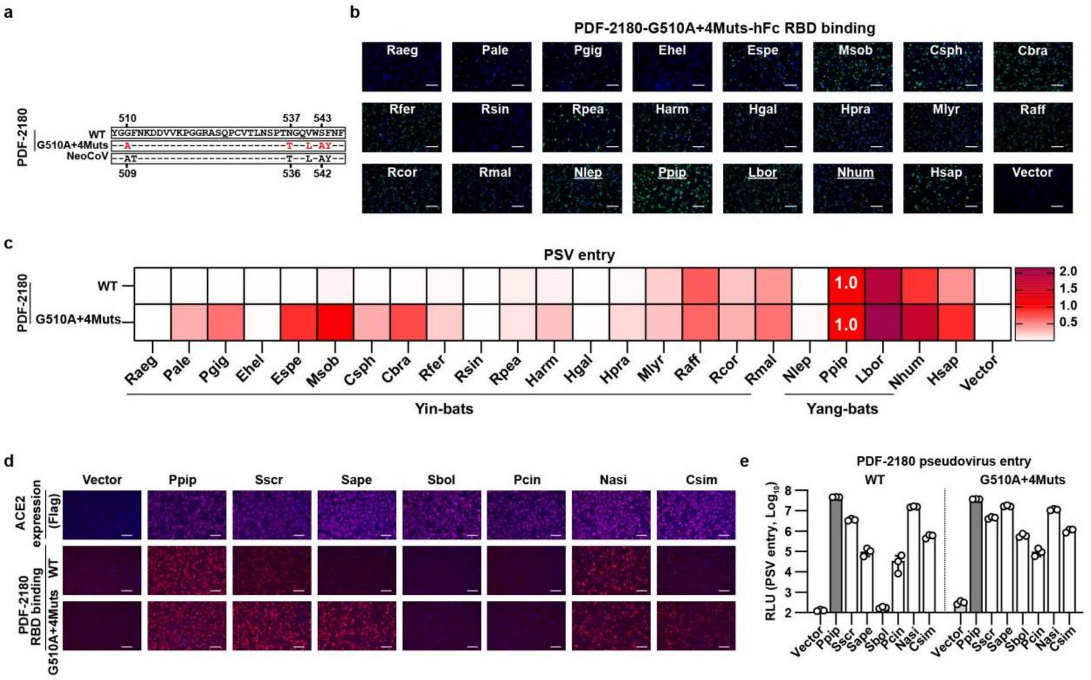

a
PDF-2180
WT
G510A+4Muts
NeoCoV
510
537
543
509
536
542
b
PDF-2180-G510A+4Muts-hFc RBD binding
Raeg
Pale
Pgig
Ehel
Espe
Msob
Csph
Cbra
Rfer
Rsin
Rpea
Harm
Hgal
Hpra
Mlyr
Raff
Rcor
Rmal
Nlep
Ppip
Lbor
Nhum
Hsap
Vector
c
PSV entry
1.0
Yin-bats
Yang-bats
2.0
1.5
0.5
d
ACE2 expression (Flag)
PDF-2180 RBD binding
Sscr
Sape
Sbol
Pcin
Nasi
Csim
e
PDF-2180 pseudovirus entry
RLU (PSV entry, Log_{10})

Supplementary Fig. S9 RBM mutation further expanded the potential host range of PDF-2180

(a) Schematic illustration of PDF-2180 swap mutants carrying the indicated NeoCoV RBD counterparts. **(b-c)** PDF-2180-G510A+4Muts-hFc RBD binding **(b)** and PDF-2180 WT & G510A+4Muts pseudoviruses entry **(c)** in HEK293T cells transiently expressing the indicated bat ACE2 orthologues**.** Entry efficiencies in PpipACE2 were set as 1.0. **(d-e)** Efficiency of PDF-2180 mutants RBD binding (**d**) and pseudovirus entry (**e**) on HEK293T cells transiently expressing the indicated mammalian ACE2 orthologues.

Data are presented as mean ± SD for n=3 biologically independent cells for **c** and **e**. Data representative of two independent experiments. RLU: relative luciferase unit. Scale bar represents 100 μm for **b** and **d**. Mw: molecular weight.
